# Routes to increase performance for antimony selenide solar cells using inorganic hole transport layers

**DOI:** 10.3389/fchem.2022.954588

**Published:** 2022-09-26

**Authors:** Stephen Campbell, Laurie J. Phillips, Jonathan D. Major, Oliver S. Hutter, Ryan Voyce, Yongtao Qu, Neil S. Beattie, Guillaume Zoppi, Vincent Barrioz

**Affiliations:** ^1^ Department of Mathematics, Physics and Electrical Engineering, Northumbria University, Newcastle Upon Tyne, United Kingdom; ^2^ Department of Physics, University of Liverpool, Liverpool, United Kingdom

**Keywords:** Sb_2_ Se_3_, photovoltaic, inorganic hole transport layers, SCAPs, thin films

## Abstract

Simple compound antimony selenide (Sb_2_Se_3_) is a promising emergent light absorber for photovoltaic applications benefiting from its outstanding photoelectric properties. Antimony selenide thin film solar cells however, are limited by low open circuit voltage due to carrier recombination at the metallic back contact interface. In this work, solar cell capacitance simulator (SCAPS) is used to interpret the effect of hole transport layers (HTL), i.e., transition metal oxides NiO and MoO_*x*_ thin films on Sb_2_Se_3_ device characteristics. This reveals the critical role of NiO and MoO_*x*_ in altering the energy band alignment and increasing device performance by the introduction of a high energy barrier to electrons at the rear absorber/metal interface. Close-space sublimation (CSS) and thermal evaporation (TE) techniques are applied to deposit Sb_2_Se_3_ layers in both substrate and superstrate thin film solar cells with NiO and MoO_*x*_ HTLs incorporated into the device structure. The effect of the HTLs on Sb_2_Se_3_ crystallinity and solar cell performance is comprehensively studied. In superstrate device configuration, CSS-based Sb_2_Se_3_ solar cells with NiO HTL showed average improvements in open circuit voltage, short circuit current density and power conversion efficiency of 12%, 41%, and 42%, respectively, over the standard devices. Similarly, using a NiO HTL in TE-based Sb_2_Se_3_ devices improved open circuit voltage, short circuit current density and power conversion efficiency by 39%, 68%, and 92%, respectively.

## 1 Introduction

Antimony selenide (Sb_2_Se_3_), as a simple and low-cost compound with a direct energy band gap (∼1.18 eV), high absorption coefficient ( $>10^{5}$ cm^−1^) and high carrier mobility (∼10 cm^2^/Vs, is a promising emergent light absorber for photovoltaic (PV) applications (Chen et al., 2015; Chen et al., 2017; Birkett et al., 2018). As a material, Sb_2_Se_3_ is mainly composed of (Sb_4_Se_6_)_*n*_ as 1-D ribbon structures, where the ribbons are strongly coupled by covalent bonds running along the c-axis with weaker Van der Waals (VdW) interactions between the ribbons. Thus, stacking of the ribbons occurs due to the weaker VdW bonds (Deringer et al., 2015). Hole mobility is enhanced in the *c*-axis and can reach 45 cm^2^/Vs along the ribbons (Black et al., 1957).

**FIGURE 1:**
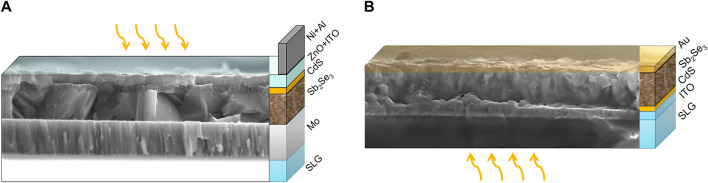
Standard planar **(A)** substrate and **(B)** superstrate configuration Sb_2_Se_3_ solar cells.

A number of studies have reported that Sb_2_Se_3_ thin films with preferred crystallographic orientation along the (*hk*1) direction, particularly (221), resulted in devices with higher efficiencies (Leng et al., 2014; Yuan et al., 2016; Li et al., 2017). The improved performance is often attributed to increased charge transport through the (*hk*1)-oriented ribbons perpendicular to the substrate and benign grain boundaries in this material (Chen et al., 2017; Williams et al., 2020). Wang and co-workers demonstrated the dependence of Sb_2_Se_3_ PV device performance on the preferred crystal orientation of the absorber (Wang et al., 2017). In that work, by optimising growth conditions, Sb_2_Se_3_ solar cells with preferred (211) and (221)-orientations on CdS and ZnO achieved higher efficiencies (5.6% and 6.0%, respectively) than those with (020) and (120)-orientations (3.2% and 4.8%, respectively). For planar Sb_2_Se_3_ solar cells in substrate orientation, a record efficiency of 6.5% has been reported with the Cd_0.75_Zn_0.25_S buffer layer being used as an alternative to CdS (Figure 1A shows standard substrate device). Meanwhile, Sb_2_Se_3_ devices with this buffer layer but in a superstrate structure (Figure 1B) have achieved an efficiency of 7.6% (Wen et al., 2018). Recently, a record substrate device efficiency of 9.2% was obtained by growing (001)-oriented Sb_2_Se_3_ nanorod arrays on sputtered molybdenum layers (Li et al., 2019). A conformal interfacial TiO_2_ layer was used to mitigate the migration of elemental antimony (Sb) into the CdS buffer layer, as interdiffusion has been shown to create a detrimental CdSe interlayer (Phillips et al., 2019).

In this work, thin transition metal oxides, NiO and MoO_*x*_, are applied as HTLs in substrate Sb_2_Se_3_ devices to improve carrier selectivity at the back electrode by controlling inter-diffusion and formation of secondary phase materials (such as MoSe_2_) at the interface. Additionally, NiO and MoO_*x*_ HTLs are deposited on superstrate Sb_2_Se_3_ films before making Au back contacts to alter the energy band alignments at the back contact effectively producing an electron reflector, and minimising carrier recombination.

In the first part of this study, Sb_2_Se_3_ substrate/superstrate device simulations using solar cell capacitance simulator (SCAPS) are conducted in order to interpret the effect of HTLs on Sb_2_Se_3_ device characteristics (Burgelman et al., 2000). We then characterise the material properties of MoO_*x*_ and NiO thin films deposited at room temperature by electron beam evaporation. At this temperature it was found that NiO formed a crystalline film, unlike MoO_*x*_ which was amorphous. Sb_2_Se_3_ absorber films were then fabricated by close-space sublimation (CSS) and thermal evaporation (TE) techniques and incorporated into superstrate and substrate solar cell configurations. HTLs were inserted at the metal electrode/Sb_2_Se_3_ absorber interface and their effect on Sb_2_Se_3_ crystallinity and solar cell performance is comprehensively studied.

## 2 Experimental section

### 2.1 Device fabrication

The basic structure of substrate Sb_2_Se_3_ solar cells was as follows: Soda lime glass (SLG)/Mo/Sb_2_Se_3_/CdS/ZnO/ITO/Ni-Al. Mo coated soda lime glass (SLG) substrates measuring 7.5 cm^2^ × 2.5 cm^2^ were used in this study. NiO or MoO_*x*_ HTLs were deposited between the Mo electrode and Sb_2_Se_3_. Thin HTL films of 15 nm thickness were deposited using e-beam evaporation. 500 nm thick Sb_2_Se_3_ layers were prepared by TE of crystalline/powder Sb_2_Se_3_ source material (Alfa Aesar, 99.99%) at a deposition rate of ∼15 Å/s. The substrates were maintained at a temperature of 300°C throughout the deposition. The Sb_2_Se_3_ films were subsequently subjected to a heat treatment at 300°C for 30 min in Ar atmosphere in a tube furnace to promote recrystallisation. For the CSS Sb_2_Se_3_ films, a compact seed layer was grown at 0.05 mbar N_2_ for 5 min with a source temperature of 350°C, followed by a 30 min growth step at 13 mbar and a source temperature of 450°C to produce a compact and highly orientated grain structure. The substrate was then rapidly cooled with N_2_. An *n*-type CdS buffer layer (∼60 nm) was deposited by chemical bath deposition followed by DC-pulsed sputtering deposition of an *i*-ZnO (∼35 nm) layer plus a transparent conductive window layer ITO (∼200 nm). Front contact grids comprising Ni (∼50 nm) and Al (∼1,000 nm) were deposited through a shadow mask by e-beam evaporation. Finally, 0.16 cm^2^ cells were defined by mechanical scribing on each substrate.

Superstrate Sb_2_Se_3_ solar cells have the following configuration: SLG/ITO/CdS/Sb_2_Se_3_/Au with NiO or MoO_*x*_ HTLs deposited between the metal contact and Sb_2_Se_3_ absorber. The ITO layer was deposited by DC-pulsed sputtering and Sb_2_Se_3_ layers were grown by TE and CSS as detailed above. Finally, Au back contacts with an area of 0.07 cm^2^ were deposited through a shadow mask by e-beam evaporation.

### 2.2 Material and device characterisation

The crystal structures of Sb_2_Se_3_ were characterised by X-ray diffraction (XRD) with Cu K*α*1 (1.54056 Å) radiation (Rigaku SmartLab SE). The surface morphology and cross-sectional images of Sb_2_Se_3_ films were taken by scanning electron microscopy (SEM, Tescan Mira 3 FEG-SEM). Optical spectroscopy measurements were performed using a Shimadzu UV-2600 spectrophotometer fitted with an integrating sphere. Kelvin probe force microscopy (KFPM) measurements were done using a KP Technology KP020 single point kelvin probe system fitted with a standard 2 mm Au tip.

Current-density vs. voltage (*J-V*) measurements of Sb_2_Se_3_ thin film solar cells were performed using an Abet Technologies solar simulator at 1-sun (100 mW/cm^2^) illumination equivalent to air mass 1.5 global spectrum with light power density calibrated using a Si reference cell.

### 2.3 Device simulation

Device simulation was carried out for both substrate and superstrate configuration Sb_2_Se_3_ solar cell using Solar Cell Capacitance Simulator (SCAPS 1-D), which is based on the solutions to Poisson’s equation and continuity equation for electrons and holes in the vertical heterostructure of multilayer thin film PV device (Burgelman et al., 2000). The input parameters of the solar cells were defined with the Sb_2_Se_3_, HTL and electron transport layer (ETL) semiconducting properties, including experimentally determined bandgaps, electron affinity, density of states (Zeng et al., 2016), mobility of charge carriers (Chen et al., 2017), acceptor/donor concentrations (Wang et al., 2015), and defect state density (Leijtens et al., 2016). Defects were introduced at the Sb_2_Se_3_/CdS interface to simulate realistic device performance.

## 3 Results and discussion

### 3.1 Simulated Sb_2_Se_3_ devices

**FIGURE 2:**
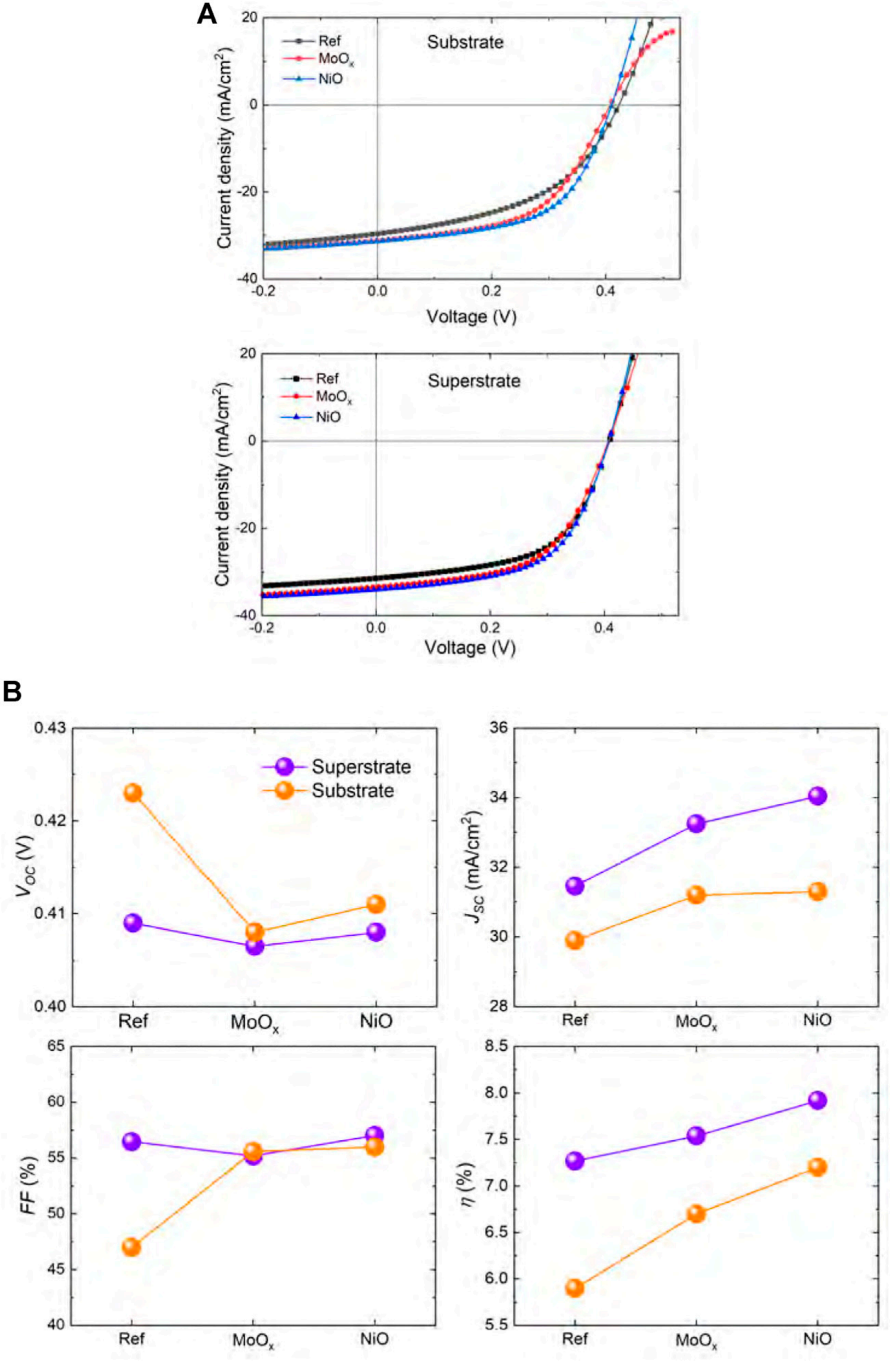
**(A)** *J-V* curves and **(B)** *J-V* parameters of simulated Sb_2_Se_3_ solar cells with different HTL materials. Roll-over behaviour is observed in the *J-V* curve of substrate devices with MoO_*x*_ HTL.

Simulation analysis using SCAPS software was implemented to evaluate the performance of reference substrate and superstrate Sb_2_Se_3_ solar cells and those incorporating MoO_*x*_ and NiO as HTLs, subsequently referred to as samples Ref, MoO_*x*_ and NiO, respectively (see Table 1 for film properties). Figure 2 shows the *J-V* curves and corresponding box plots of *J-V* parameters of both Sb_2_Se_3_ device configurations with incorporated HTLs. Regarding the substrate devices, all device parameters are improved, with the exception of *V*_*oc*_ which shows a slight decrease for devices with a HTL (down from 0.423 V for the reference device to 0.408 and 0.411 V for MoO_*x*_ and NiO devices, respectively). However, devices with MoO_*x*_ HTL show evidence of roll-over behaviour. The roll-over phenomenon, which occurs near the *V*_*oc*_ in a light *J-V* curve, is due to Schottky energy barrier formed at the absorber/metal interface at a solar cell back contact (Eisenbarth et al., 2011; Hädrich et al., 2011). It acts as a reverse biased diode when the main junction is forward biased, blocking carrier transport for increasing forward bias, resulting in roll-over behaviour in light *J-V* characteristics. The baseline *J*_*sc*_ in the reference device was 29.9 mA/cm^2^, rising to 31.2 and 31.3 mA/cm^2^ in MoO_*x*_ and NiO devices, respectively. Addition of HTL films to the reference device demonstrated a notable increase in *FF* for substrate devices. The *FF* in the reference device was 47.0%, rising to a maximum of 55.6% and 56.0% in the MoO_*x*_ and NiO devices, respectively. The increase in *J*_*sc*_ and *FF* of devices with integrated HTL materials directly translates into improvements in power conversion efficiency, PCE [*η* = 5.9% (Ref), 6.7% (MoO_*x*_) and 7.2% (NiO)]. The current-blocking energy barrier at the back contact of the MoO_*x*_ substrate device could explain the lower PCE in comparison to the device with a NiO HTL. It is important to note that the results shown are not representative of the maximum conversion efficiencies that may be achieved with Sb_2_Se_3_, as we are focusing solely on the effect of the HTL, while using currently available materials parameters.

**FIGURE 3:**
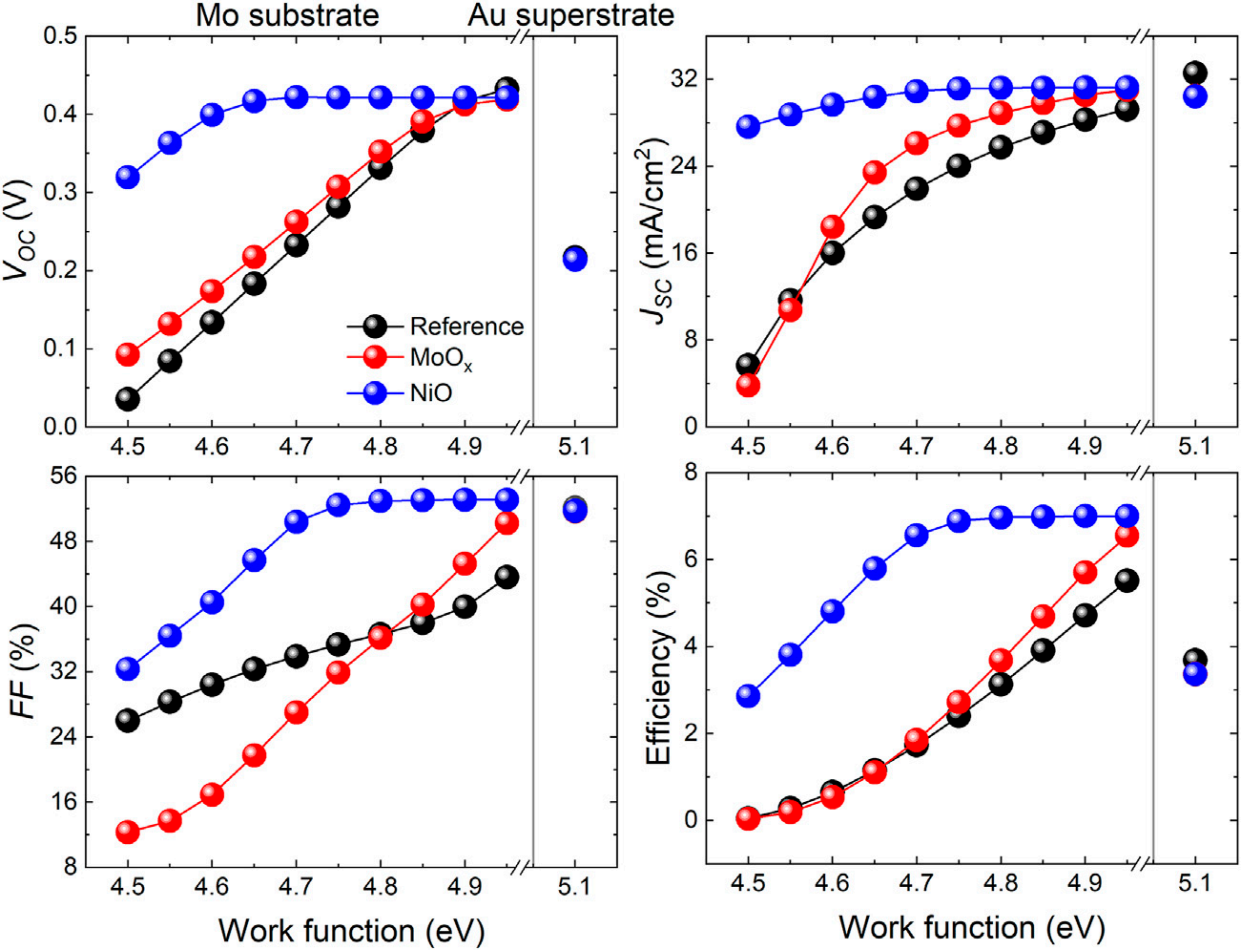
*J-V* parameters of simulated Sb_2_Se_3_ substrate devices with Mo back contact (varying Mo WF between 4.50–4.95 eV) and simulated Sb_2_Se_3_ superstrate devices with Au back contact (WF at 5.1 eV).

For superstrate Sb_2_Se_3_ solar cells, devices with an incorporated HTL showed an increase in *J*_*sc*_ of around 8% from 31.5 mA/cm^2^ observed in the reference device to 33.5 and 34.0 mA/cm^2^ in the devices with a MoO_*x*_ and NiO HTL, respectively. As a result of the improvement in *J*_*sc*_, the PCE of solar cells with a HTL increased to 7.5% (MoO_*x*_) and 8.0% (NiO) from the reference value of 7.3%. Interestingly, no roll-over was seen in the *J-V* curve for the MoO_*x*_ device which could be related to the use of Au as metallic back contact rather than Mo in the substrate devices. The work function (WF) of a metal employed as a rear contact on a PV device plays an important role in facilitating hole extraction at the contact (Fleck et al., 2020). Typically, Au is reported to have a WF of 5.10 eV (Michaelson, 1977) and Mo has WFs ranging from 4.50–4.95 eV, depending on the preferred crystal orientation of the metal (Green, 1969; Michaelson, 1977; Hölzl and Schulte, 1979). To illustrate the effect of back contact metal WF on substrate/superstrate Sb_2_Se_3_ device performance, Figure 3 shows the dependence of *J-V* parameters on the WF of Mo and Au metals. It is apparent that the J-V parameters of all substrate devices are sensitive to variations in the value of Mo WF. In the Ref and MoO_*x*_ substrate devices, *V*_*oc*_ decreases monotonically with Mo WF where a significant drop is observed from 0.432 V to 0.422 V at WF 4.95 eV to 0.036 V and 0.093 V at WF 4.50 eV for Ref and MoO_*x*_ devices, respectively. This is a clear indication of an increasing back contact barrier with decreasing Mo WF. This phenomenon has been observed experimentally in Sb_2_Se_3_ solar cells previously (Liu et al., 2014; Li et al., 2017). The *V*_*oc*_ in the NiO device is less affected by the Mo WF, reducing from 0.422 V at WF 4.95 eV to 0.319 V at WF 4.50 eV. A similar trend is seen in *J*_*sc*_, *FF* and *η* parameters for the substrate devices. However, a low Mo WF of 4.50 eV causes a notable decrease in *FF* of the MoO_*x*_ device (12.3%), compared to the Ref and NiO devices (26.0% and 32.4%).

**FIGURE 4:**
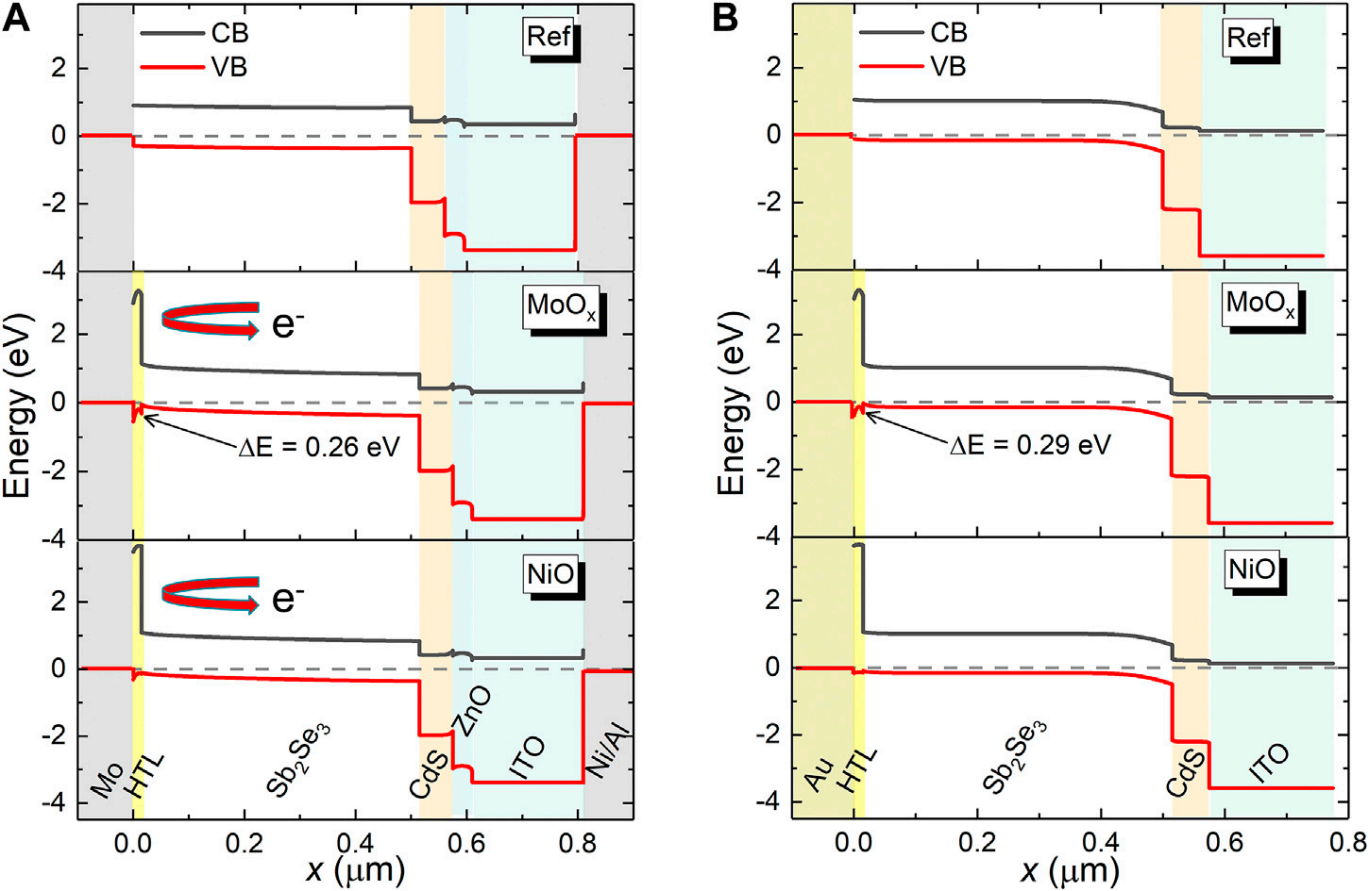
Energy level alignment for the devices in substrate **(A)** and superstrate **(B)** orientati1ons. Devices without a hole transport layer (top), with a MoO_*x*_ layer (middle) and a NiO layer (bottom) are shown.

In order to understand the improvement of the device performance with the introduction of HTLs, it is necessary to consider the energy band alignment at the interfaces at the back of the PV devices. Figure 4 shows the simulated energy band diagrams of substrate and superstrate Sb_2_Se_3_ devices incorporating NiO and MoO_*x*_ HTLs. Due to a small electron affinity (EA = 1.46 eV (NiO), 2.05 eV (MoO_*x*_)) and large band gaps (*E*_*g*_ ∼3.80 eV (NiO), 3.50 eV MoO_*x*_x)) in both HTL materials, a large potential energy barrier is formed at the back contact, reflecting electrons. This barrier minimises carrier recombination at the back interfaces with Sb_2_Se_3_ and improves conductivity at the back electrode. However, it is apparent that a non-negligible hole barrier of 0.26 and 0.29 eV is formed at the MoO_*x*_/Sb_2_Se_3_ interface of the substrate and superstrate devices, respectively, which can manifest as *J-V* roll-over behaviour seen in the simulated MoO_*x*_ substrate device. Thus the SCAPS simulations indicate the incorporation of a MoO_*x*_ or NiO HTL into substrate and superstrate configuration Sb_2_Se_3_ solar cells increases device performance compared to a standard solar cell by the introduction of a high energy barrier to electrons at the rear absorber/metal interface.

### 3.2 Fabricated Sb_2_Se_3_ devices

**FIGURE 5:**
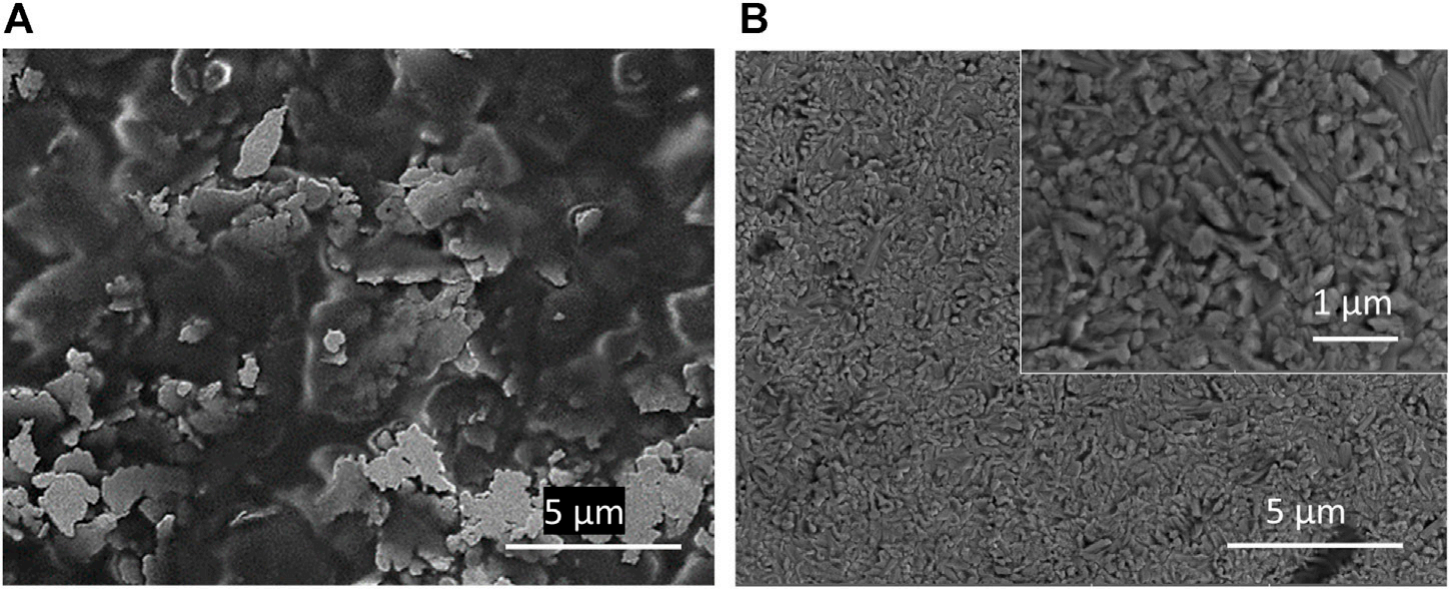
Top-down SEM image of a 100 nm **(A)** MoO_*x*_ film and **(B)** NiO films on glass. Inset: Higher magnification image of the NiO film, showing the nanostructure.

**FIGURE 6:**
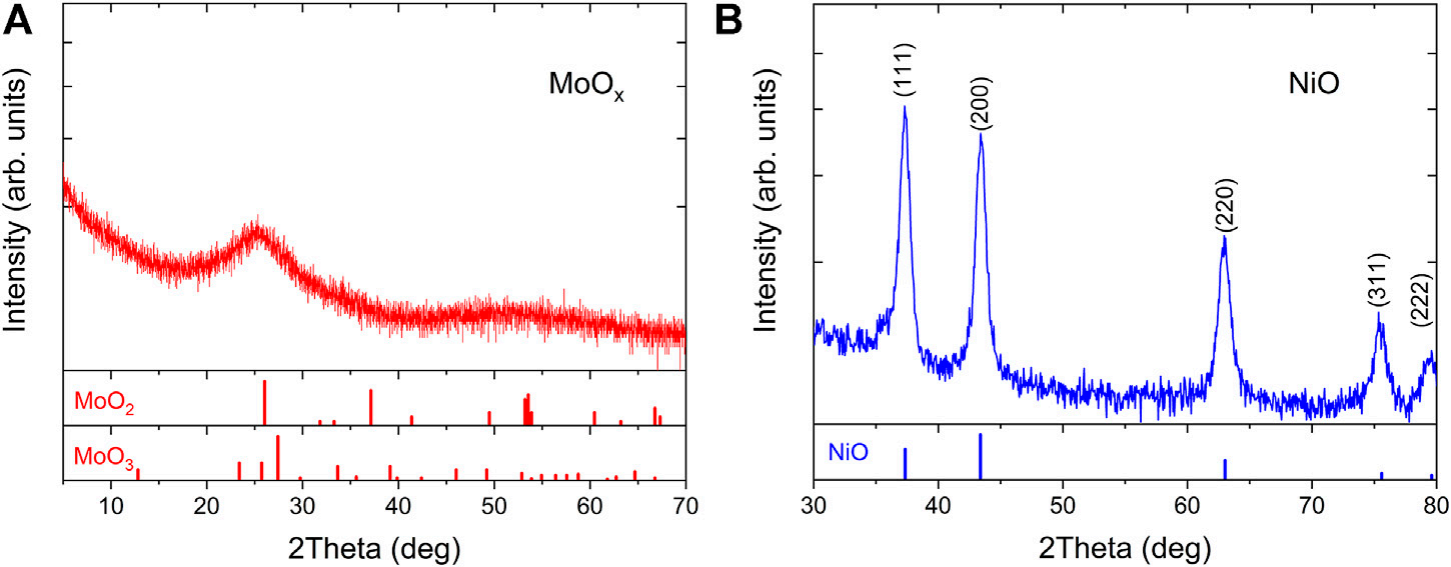
XRD pattern of 100 nm films of **(A)** MoOx and **(B)** NiO on soda lime glass (SLG). Reference XRD data for MoO_2_, MoO_3_ and NiO are shown underneath the XRD with JPDCS card ID 65-5787, 35-0609 and 04-0,835 respectively.

100 nm thick films of MoO_*x*_ and NiO were deposited on SLG at room temperature to facilitate characterisation of the HTLs. Figure 5 shows surface morphology SEM images of the respective HTLs. The MoO_*x*_ film exhibits an amorphous, flake-like structure in comparison to a compact crystalline morphology observed in the NiO film. XRD patterns in Figure 6 confirm the amorphous and crystalline nature of the MoO_*x*_ and NiO films, respectively. All the diffraction peaks in the NiO thin film were identified and indexed to cubic NiO (JCPDS number 04-0835) and no diffraction peaks of other impurity phases were observed.

Supplementary Materials S1A shows the spectral transmittance and reflectance of the NiO and MoO_*x*_ films on SLG. Both HTLs are highly transparent in the visible and near-infrared wavelength region and their transmittance falls sharply at ultraviolet wavelengths. However, the amorphous MoO_*x*_ film has slightly lower transmittance/higher reflectance in the sub-600 nm wavelength region compared to the crystalline NiO film. The bandgap energy (*E*_*g*_) of the HTL films was calculated by extrapolation of the linear region of the Tauc plot to the *x*-axis, according to the relation (Tauc et al., 1966):

$${\left(\alpha h\nu \right)}^{2}=A\left(h\nu -E_{g}\right)$$ (1)

where *α* is the absorption coefficient of the semiconductor material, *h* is Planck’s constant, *ν* is the frequency of the electromagnetic radiation and A is a constant of proportionality. The estimated *E*_*g*_ values of NiO and MoO_*x*_ films are 3.95 and 3.85 eV, respectively (see Supplementary Materials S1B). A HTL film thickness of 15 nm was incorporated into the superstrate/substrate device to ensure a conformal coating of the HTL. A HTL requires a thickness sufficient to preserve the desired material properties and not impede charge transport considerably which would detrimentally increase series resistance in the finished devices.

### 3.3 Superstrate devices

**FIGURE 7:**
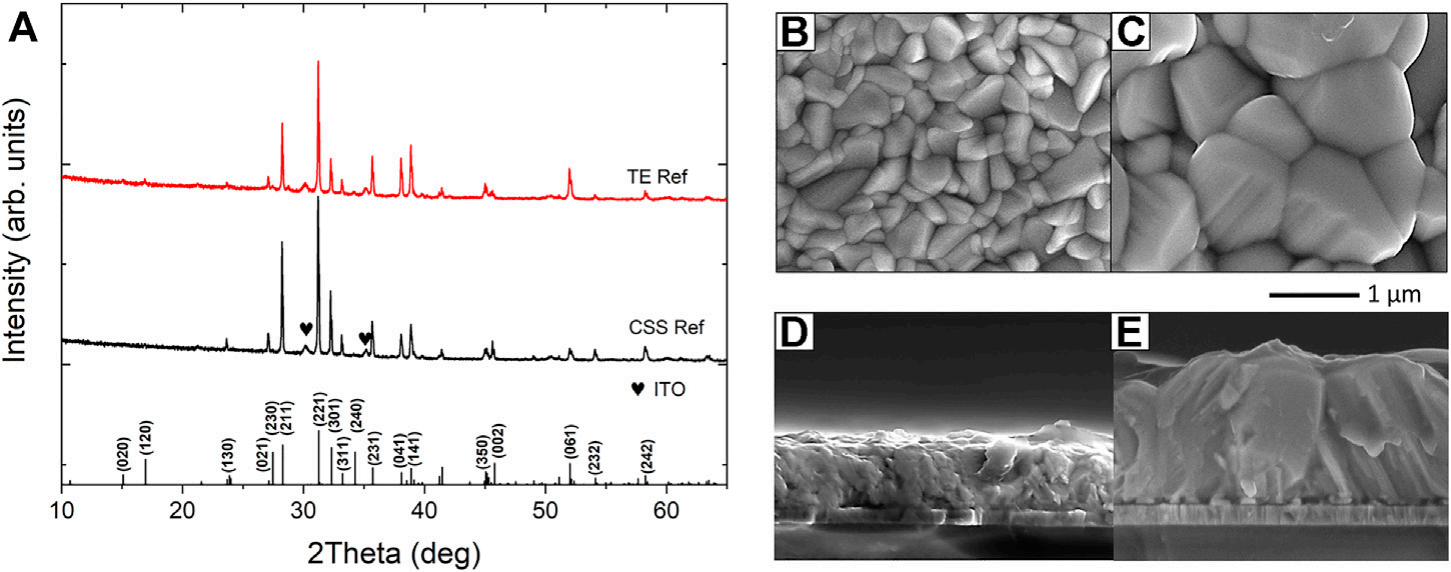
**(A)** XRD patterns of Sb_2_Se_3_ layers deposited by TE and CSS on ITO/CdS superstrates with standard diffraction pattern for Sb_2_Se_3_ (JCPDS15-0861) included for reference and SEM images of corresponding TE **(B,D)** and CSS **(C,E)** Sb_2_Se_3_ samples.

TE and CSS deposition techniques were employed for Sb_2_Se_3_ film growth on SLG/ITO/CdS superstrates. For TE, the SLG/ITO/CdS superstrates were heated to 300°C prior to Sb_2_Se_3_ deposition in order to promote the growth of preferred (*hk*1) crystal orientations while minimising (*hk*0) orientations (Zhou et al., 2015) (*hk*0) planes, specifically (120), have been found to be detrimental to carrier transport (Guo et al., 2018; Wen et al., 2018; Li et al., 2019). The (*hk*0)-oriented Sb_2_Se_3_ nanoribbons are stacked parallel to the ITO/SLG superstrate where conductivity is inhibited by electrically insulating VdW bonds between the stacked nanoribbons. A seed layer is used in Sb_2_Se_3_ films deposited *via* CSS. This seed layer has a high density of nucleation points for the second stage of growth during the CSS process, which improves uniformity, raising the average efficiency of devices (Hutter et al., 2018a). Transmittance and reflectance data for a representative TE Sb_2_Se_3_ film was used to determine the *E*_*g*_ from a Tauc plot, which gave a *E*_*g*_ value of 1.17 eV in good agreement with (Birkett et al., 2018), see Supplementary Materials S2A,B. XRD patterns for Sb_2_Se_3_ films deposited by TE and CSS are shown in Figure 7A. The peaks in both XRD patterns are sharp and well resolved indicating the polycrystalline nature of the Sb_2_Se_3_ thin films. The lattice planes are cross-referenced to JCPDS card no. 15-0861 confirming the formation of orthorhombic Sb_2_Se_3_ with space group Pbnm. Both XRD patterns show similar characteristics, exhibiting strong (211) and (221) peaks with minimal contributions from (*hk*0) planes. Figures 7B–E shows the top and cross-sectional SEM images of Sb_2_Se_3_ thin films deposited by TE and CSS. The different growth techniques result in contrasting Sb_2_Se_3_ film morphologies. TE produces Sb_2_Se_3_ films of uniform thickness of ∼500 nm and densely packed grains, confirming the good crystallinity of the films, consistent with the XRD results (Figures 7B,D). However, this deposition method did not form a conformal coating of the Sb_2_Se_3_ film across the entire superstrate with the presence of pinholes observed, see Supplementary Materials S3A.

Conversely, CSS-grown Sb_2_Se_3_ films have a rough surface morphology with exceptionally large grains in comparison to the TE films and the grains extend the full depth of the layer. Larger grains are a prerequisite for better device performance as charge mobility is faster along the Sb_2_Se_3_ ribbons than hopping between the ribbons (see Figures 7C,E). The CSS films also showed a degree of porosity but not to the extent observed in the TE films, Supplementary Materials S3B. The presence of pinholes in the Sb_2_Se_3_ films is detrimental to device performance as shunting pathways may be formed upon subsequent deposition of the Au back contact (Hutter et al., 2018b).

**FIGURE 8:**
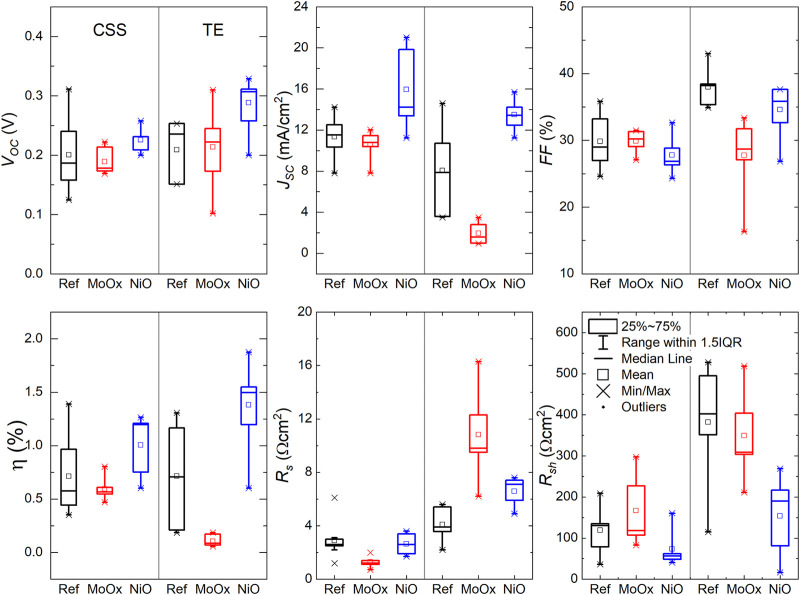
*J-V* parameters of superstrate TE and CSS Sb_2_Se_3_ devices with incorporated MoO_*x*_ and NiO HTLs. □ is the average value and × is the minimum and maximum position. The three horizontal lines of each box stand for the 25%, 50%, and 75% of the reading distribution. The whisker range is determined by the standard deviation of the sampled devices. IQR is the inter-quartile range.

*J-V* measurements under 1-sun illumination (100 mW/cm^2^) were performed on Sb_2_Se_3_ devices in the standard superstrate configuration and devices incorporating MoO_*x*_ and NiO HTLs. The light *J-V* curves were fitted using a single diode model to extract the values of series (*R*_*s*_) and shunt (*R*_*sh*_) resistances. Figure 8 compares the statistical distribution of the key PV parameters for these devices, where a minimum of 10 cells of each device type were measured. On average, there was a slight increase in *V*_*oc*_ when a NiO HTL was incorporated into the CSS device structure. Using a NiO HTL layer increased *V*_*oc*_ to 0.226 V from values of 0.201 and 0.186 V for Ref and MoO_*x*_ devices, respectively. The mean *J*_*sc*_ of NiO cells was also enhanced to 15.94 mA/cm^2^ compared to Ref (11.34 mA/cm^2^) and MoO_*x*_ (10.54 mA/cm^2^) cells despite a slightly lower average *FF* in the NiO devices. This translates into a higher mean NiO CSS device efficiency of 1.01% with Ref and MoO_*x*_ devices achieving efficiencies of 0.71 and 0.59% respectively. Notwithstanding the higher average *R*_*s*_ (2.6 Ωcm^2^) and lower *R*_*sh*_ (74 Ωcm^2^) values for NiO CSS solar cells compared to Ref (*R*_*s*_ = 2.9 Ωcm^2^, *R*_*sh*_ = 119 Ωcm^2^) and MoO_*x*_ (*R*_*s*_ = 1.3 Ωcm^2^, *R*_*sh*_ = 167 Ωcm^2^) cells, using NiO as a HTL increases performance by boosting *J*_*sc*_ in CSS Sb_2_Se_3_ superstrate devices compared to the standard and MoO_*x*_ based devices.

The average *J-V* parameters of TE Sb_2_Se_3_ superstrate devices followed a similar trend to those observed in the CSS devices [*V*_*oc*_: 0.209 V (Ref) → 0.214 V (MoO_*x*_) → 0.288 V (NiO), *J*_*sc*_: 1.94 mA/cm^2^ (MoO_*x*_) → 8.05 mA/cm^2^ (Ref) → 13.48 mA/cm^2^ (NiO) ⇒ *η*: 0.10% (MoO_*x*_ → 0.72% (Ref) → 1.38% (NiO)]. It is worth noting that the mean *FF* of the Ref TE cells (38.0%) was higher in relation to the cells with a HTL (27.2% MoO_*x*_, 34.6% NiO). This correlates to an increase in *R*_*sh*_ of 382 Ωcm^2^ in Ref samples from *R*_*sh*_ values of 349 Ωcm^2^ and 154 Ωcm^2^ measured in MoO_*x*_ and NiO cells, respectively. In TE superstrate device configuration, the thin MoO_*x*_ film appears to form a more resistive layer compared to Ref and NiO devices (*R*_*s*_: 10.8 Ωcm^2^ MoO_*x*_, 4.1 Ωcm^2^ Ref and 6.6 Ωcm^2^ NiO). Thus, overall device performance in MoO_*x*_ based solar cells is negatively impacted by low *J*_*sc*_ and high *R*_*s*_ which could be related to the amorphous nature of the MoO_*x*_ thin film and the presence of a current-blocking barrier at the back contact highlighted in device simulations. Despite lower *FF* in NiO based solar cells, device efficiencies exceed those of Ref and MoO_*x*_ TE devices due to improvements in *V*_*oc*_ and *J*_*sc*_ showing the benefit of using NiO as a HTL in superstrate Sb_2_Se_3_ solar cells.

### 3.4 Substrate devices

**FIGURE 9:**
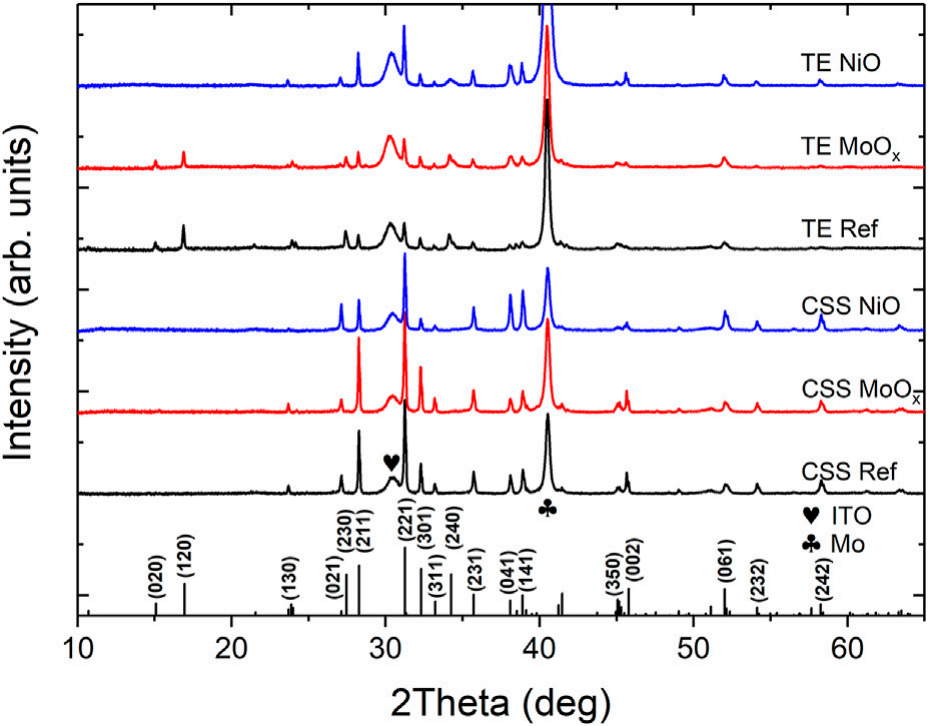
XRD patterns of Sb_2_Se_3_ films deposited by TE or CSS on top of NiO, MoO_*x*_ and Mo-coated SLG.

Figure 9 shows the XRD patterns of substrate Sb_2_Se_3_ thin films deposited *via* TE and CSS. All diffraction peaks are in good agreement with the orthorhombic Sb_2_Se_3_ (JCPDS 15-0861), which presents in the form of (*hk*0), (*hk*1) or (*hk*2). No diffraction peaks of other impurity phases were observed. TE Sb_2_Se_3_ films on Mo and Mo/MoO_*x*_ substrates show (020) and (120) peaks compared to all other Sb_2_Se_3_ films. The presence of (020) and (120) crystal orientations in thin Sb_2_Se_3_ films adversely affects PV device performance (Leng et al., 2014; Yuan et al., 2016; Li et al., 2017). However, when using a NiO HTL in TE Sb_2_Se_3_ films, it can be observed that the intensity of the diffraction peaks of Sb_2_Se_3_ is dominated by (221) and (211) crystal plane orientations. Furthermore, when using the Mo/NiO substrate, Sb_2_Se_3_ film shows an increased peak intensity for the (002) orientation. Since *h* and *k* miller indices have a zero value, it indicates that the (Sb_4_Se_6_)_*n*_ ribbons grow perpendicular to the substrate surface (Li et al., 2019). For CSS Sb_2_Se_3_ films, Ref and MoO_*x*_ samples demonstrate a higher (002) peak intensity than NiO.

**FIGURE 10:**
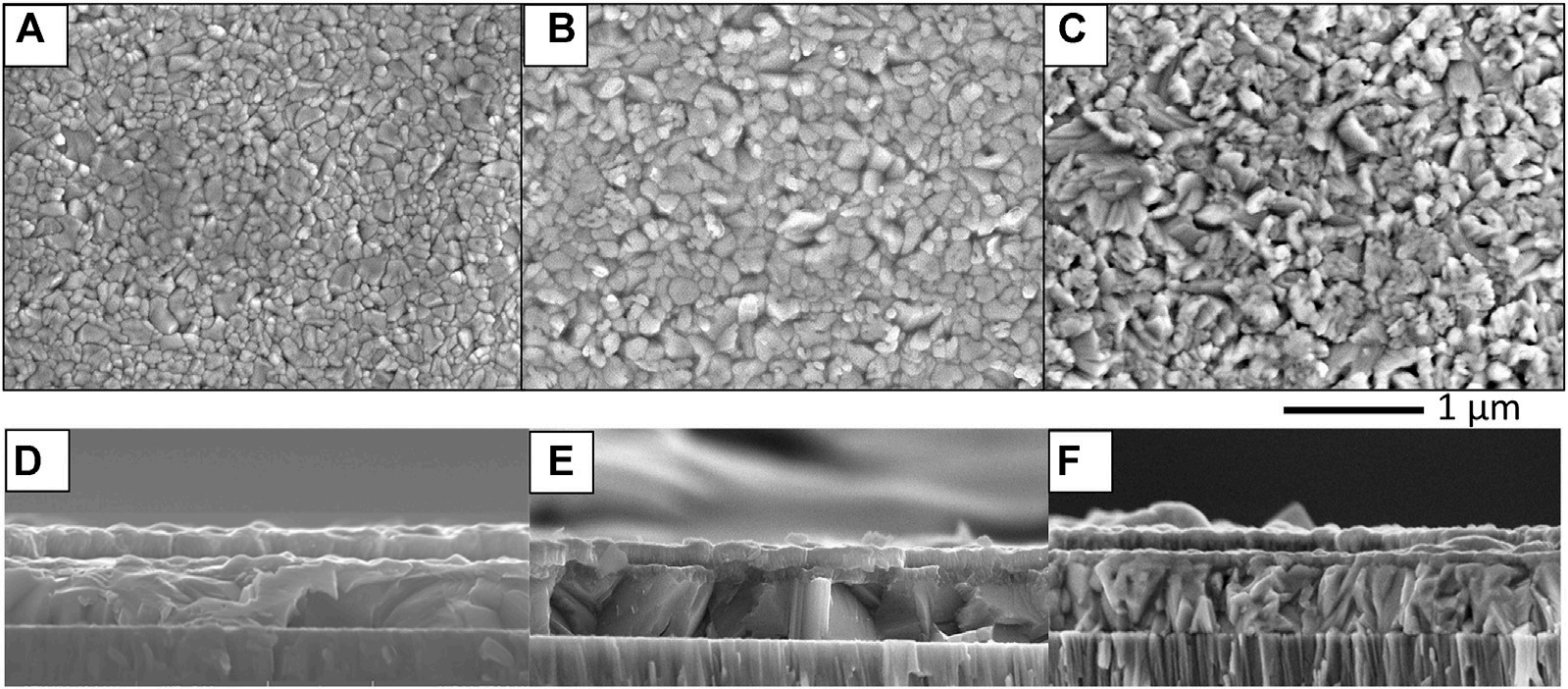
Top-down and cross-sectional SEM images of reference substrate **(A,D)**, MoO_*x*_ **(B,E)** and NiO **(C,F)** of Sb_2_Se_3_ films deposited by thermal evaporation.

**FIGURE 11:**
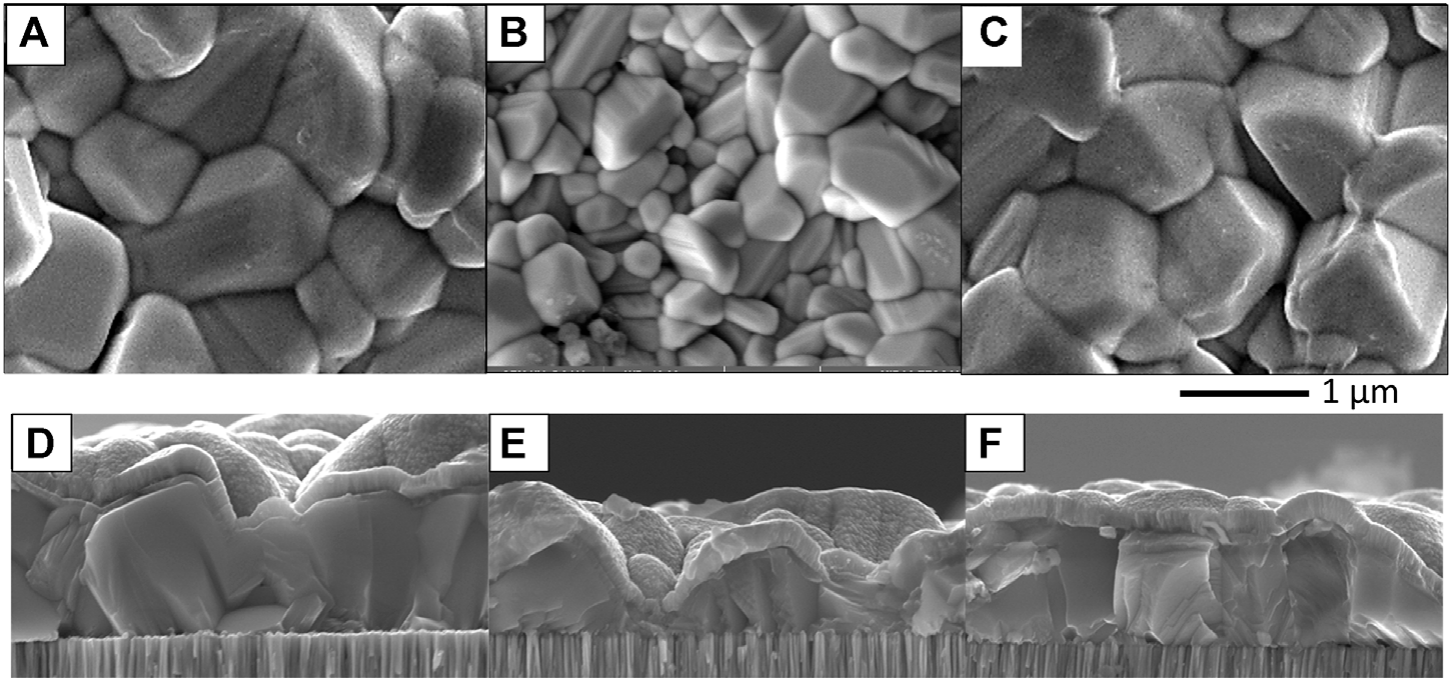
Top-down and cross-sectional SEM images of reference substrate **(A,D)**, MoO_*x*_ **(B,E)** and NiO **(C,F)** of Sb_2_Se_3_ films deposited by close-space sublimation.

Figures 10, 11 show SEM images of Sb_2_Se_3_ films on Mo-coated SLG deposited by TE and CSS methods, respectively. The top-down SEM images of the TE films (Figures 10A–C) show a difference in morphology depending on the presence of the underlying HTL. The MoO_*x*_ sample exhibits larger Sb_2_Se_3_ grains than the Ref sample and the presence of pinholes in both samples is patently obvious. On the other hand, the Sb_2_Se_3_ grains in the NiO sample appear more angular in nature although pinholes are still present in the film. The dissimilarity in morphology is emphasised in SEM cross-section images of the TE Sb_2_Se_3_ films (Figures 10D–F). Voids at the absorber/Mo interface are apparent in the Ref TE sample whereas the MoO_*x*_ sample shows a homogenous film with large grains. For the NiO sample, the Sb_2_Se_3_ grains appear column-like with no voids at the Mo interface. The top-down SEM image of all types of CSS Sb_2_Se_3_ thin films (Figures 11A–C) show significantly larger grains compared to the TE films. However, Sb_2_Se_3_ film in the Ref sample is on average thicker (∼1,000 nm) than the MoO_*x*_ (∼550 nm) and NiO (∼700 nm), see Figures 11D–F. The NiO sample also has a smoother surface topography.

**FIGURE 12:**
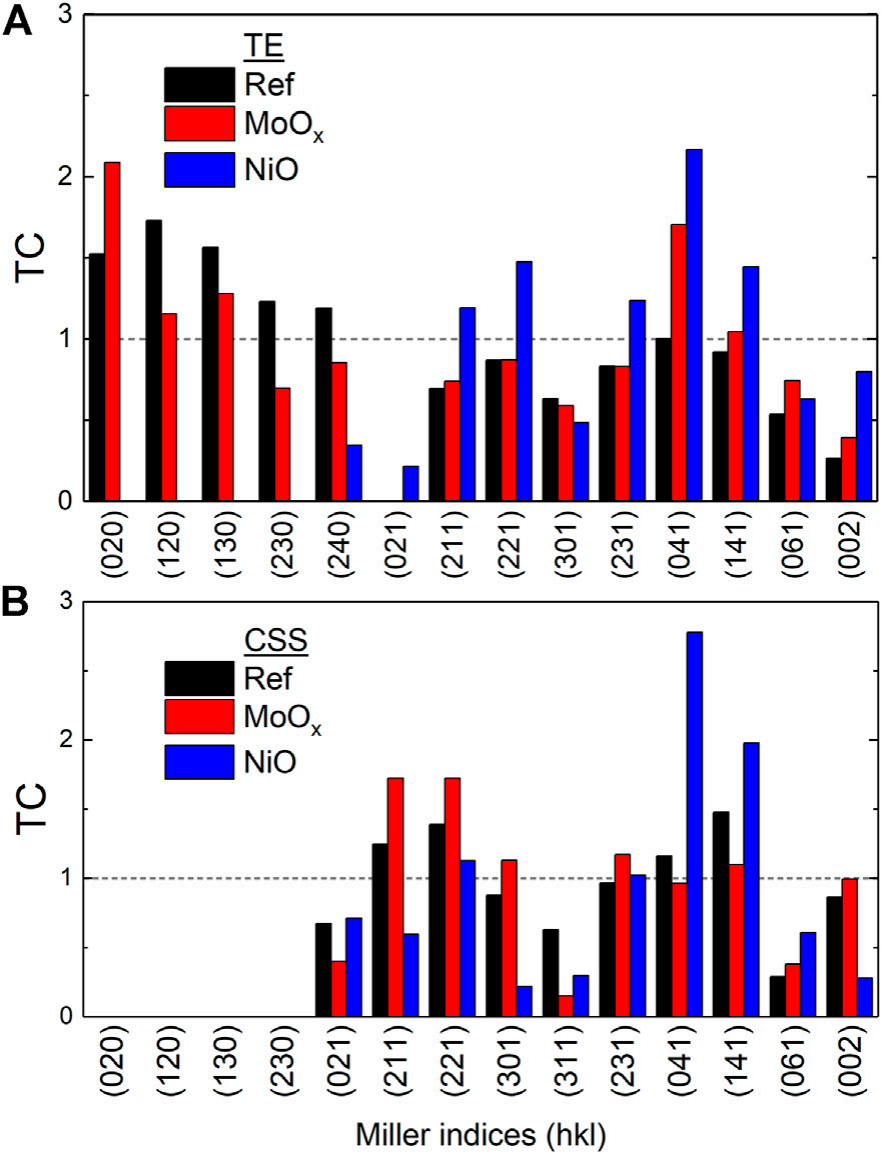
Texture coefficient analysis from XRD patterns of Sb_2_Se_3_ films deposited *via* TE **(A)** and CSS **(B)** with different hole transport layers in substrate configuration. A diffraction peak with a relatively large TC value ( > 1) indicates a preferred orientation of the grain along this direction.

To quantify the difference in orientations between the substrate Sb_2_Se_3_ thin films, the texture coefficient (TC) of diffraction peaks of the samples was calculated based on the following equation (Zoppi et al., 2006):

$$TC_{\left(hkl\right)}=\frac{\frac{I_{\left(hkl\right)}}{I_{0}\left(hkl\right)}}{\frac{1}{N}\sum_{N}\frac{I_{\left(hkl\right)}}{I_{0}\left(hkl\right)}}$$ (2)

where *I*_(*hkl*)_ is the measured peak intensity of (*hkl*) plane and *I*_0_ (*hkl*) the intensity in the standard XRD pattern. *N* is the total number of reflections considered for the calculation. A diffraction peak with a relatively large TC value ( > 1) indicates a preferred orientation of the grain along this direction. Figure 12 shows the TC for Sb_2_Se_3_ thin films with HTLs deposited by (A) TE and (B) CSS. It is apparent from Figure 12 that NiO HTL plays a critical role in eliminating the detrimental (*hk*0) planes in the TE samples and at the same time, significantly increases absorber growth in planes, i.e., (211), (221) that are perpendicular to the substrate surface. This further supports the enhanced device performance in solar cells when NiO is used as the HTL. In CSS samples, this templating effect of HTLs is not observed as no (*hk*0) planes are grown in the Ref and MoO_*x*_ samples. MoO_*x*_ increases the growth of favoured crystal planes including (211), (221), and (002) compared to the Ref substrate sample whereas NiO appears to inhibit the growth of the preferential planes, which may be attributed to rendering the seed layer ineffective but further study will be required to fully understand the reason.

**FIGURE 13:**
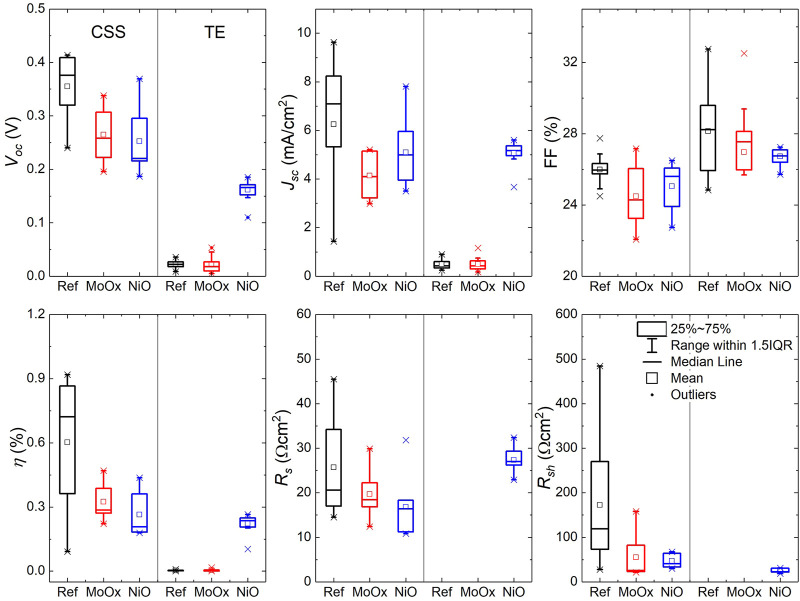
*J-V* parameters of substrate TE and CSS Sb_2_Se_3_ devices with incorporated MoO_*x*_ and NiO HTLs. □ is the average value and × is the minimum and maximum position. The three horizontal lines of each box stand for the 25%, 50% and 75% of the reading distribution. The whisker range is determined by the standard deviation of the sampled devices. IQR is the inter-quartile range.

Figure 13 shows the variation in *J-V* parameters measured for a minimum batch size of 10 Sb_2_Se_3_ solar cells in substrate configuration deposited by TE and CSS incorporating HTLs. The use of MoO_*x*_/NiO HTLs adversely affects all device parameters in CSS-based solar cells. This can be explained by lower average *R*_*sh*_ values of 55 Ωcm^2^ and 47 Ωcm^2^ determined for MoO_*x*_ and NiO device types, respectively, compared to 172 Ωcm^2^ in the Ref devices. The reason for the reduction in *R*_*sh*_ of the substrate devices with a HTL is not obvious. Only working TE devices were achieved by incorporating a NiO HTL, which can be attributed to the templating effect of the NiO film which eliminated the deleterious (*hk*0) crystal planes and promoted the growth of preferred (211) and (221) planes. As highlighted in device simulations, the performance of substrate Sb_2_Se_3_ solar cells can be dependent on the WF of Mo back contact (see Figure 3). Mo metal typically has a WF in the range of 4.5–4.95 eV. KPFM measurements on Mo coated SLG prior to Sb_2_Se_3_ deposition determined the Mo WF to be 4.6 eV. According to simulations, device performance of Ref and MoO_*x*_ substrate devices is severely impacted at the observed Mo WF. Simulated NiO device performance is affected to a lesser degree.

*R*_*s*_ values for both TE and CSS substrate Sb_2_Se_3_ devices were significantly higher than their superstrate counterparts and had a detrimental effect on overall substrate device performance. This could be related to a non-optimal sputtered ITO layer in the substrate devices with a typical sheet resistance of ∼ 35 Ω/□ (Qu et al., 2016) compared to commercially available ITO-coated glass slides used in superstrate devices with sheet resistances of 8–12 Ω/□ (Sigma Aldrich).

### 3.5 Simulated and fabricated device comparison

**FIGURE 14:**
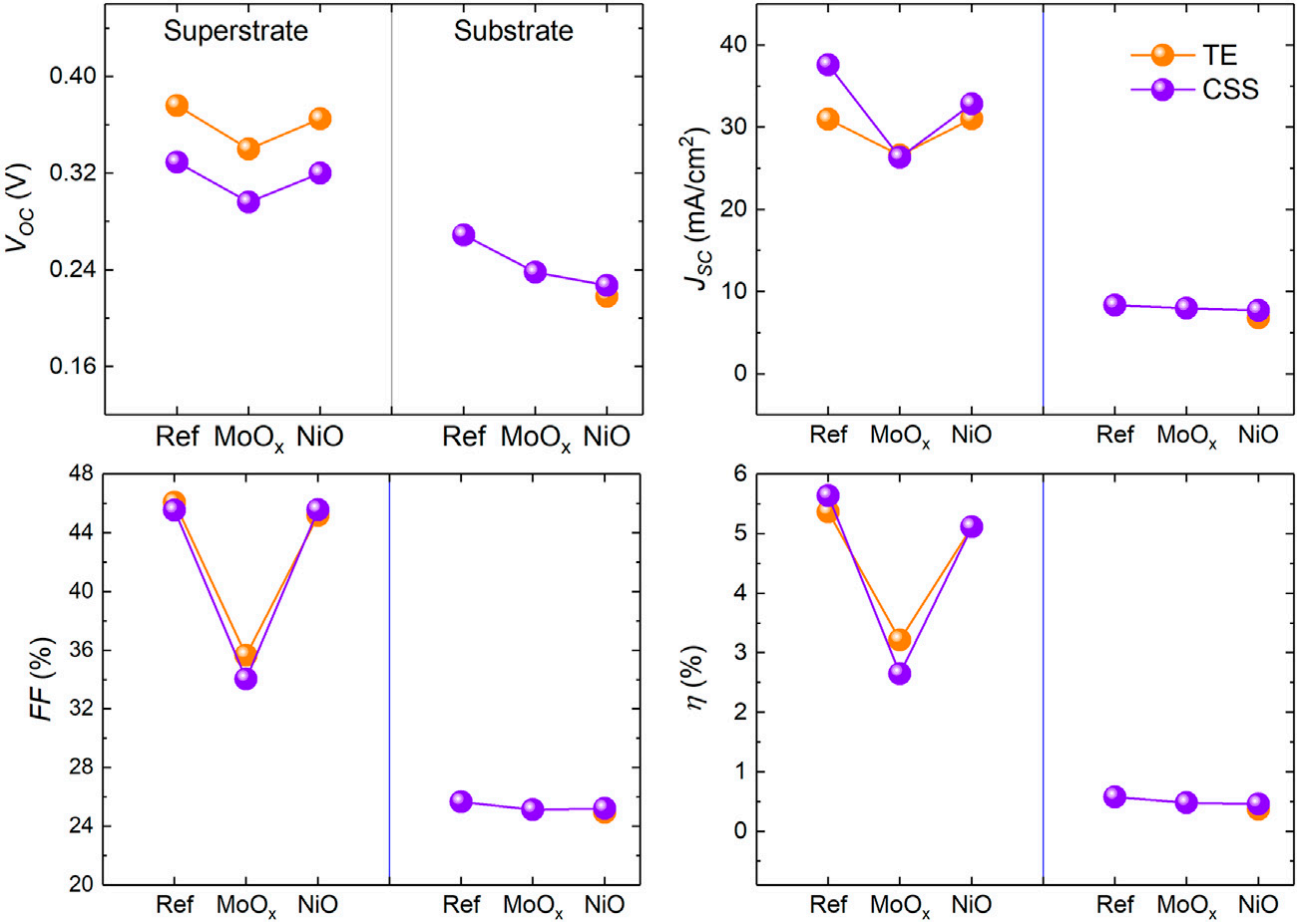
Comparison of *J-V* parameters of simulated TE and CSS Sb_2_Se_3_ solar cells with different HTL materials in substrate and superstrate device configurations.

Experimentally determined device parameters, such as *R*_*s*_ and *R*_*sh*_ and apparent doping density (*N*_*A*_) of the Sb_2_Se_3_ absorber, were incorporated into SCAPS simulations of TE/CSS Sb_2_Se_3_ devices in superstrate/substrate configurations in order to replicate the observed behaviour of the fabricated devices. For an accurate representation of the fabricated cells, the *N*_*A*_ value for the Sb_2_Se_3_ absorber in the CSS devices was set to a value previously determined for the same CSS deposition process used in this study with a Sb_2_Se_3_ absorber thickness of 1 *μ*m (Phillips et al., 2019). An experimentally determined *N*_*A*_ value for a typical 500 nm thick TE Sb_2_Se_3_ absorber was used in TE device simulations (see Table 1 for TE/CSS Sb_2_Se_3_ film properties). Figure 14 shows device performance of the simulated TE/CSS Sb_2_Se_3_ devices with experimentally determined *R*_*s*_, *R*_*sh*_ and *N*_*A*_ values. Similar trends are observed for all device parameters of the simulated and fabricated solar cells in both device configurations indicating the simulated devices are a reasonable representation of actual Sb_2_Se_3_ solar cells (see Figures 8, 13). However, in superstrate configuration, simulations overestimate all *J-V* parameters, indicating factors other than *R*_*s*_, *R*_*sh*_ and *N*_*A*_ are influencing device performance. Material properties such as carrier lifetimes, defects and band tails states have been cited as having a detrimental effect on overall device performance (Chen and Tang, 2020). In that work, a number of bulk defects in Sb_2_Se_3_ were identified with energy levels within the Sb_2_Se_3_ bandgap ranging from 0.18–0.94 eV above the valence band maximum. For simulation purposes, a mid-gap donor defect (0.62 eV) was introduced for the Sb_2_Se_3_ bulk to reproduce realistic device performance (Wen et al., 2018; Ma et al., 2020). Chen and Tang (2020) also highlighted significant recombination occurring at the *n-p* interface which severely impacts both *V*_*oc*_ and *J*_*sc*_. The presence of additional Sb_2_Se_3_ bulk defects and increased absorber/buffer interface defect concentration could account for the differences observed between the simulated and fabricated devices studied here.

**TABLE 1:** Device simulation parameters, *d*: layer thickness, *E*_*g*_: bandgap, *χ*: electron affinity, *ɛ*/*ɛ*_0_: dielectric constant, *N*_*C*/*V*_: effective density of states C: conduction band (CB) V: valence band (VB), *μ*_*e*,*h*_: carrier mobility, *N*_*A*/*D*_: apparent doping density D: donor A: acceptor, *σ*_*e*,*h*_: capture cross section, *N*_*int*_: interface defect concentration, *E*_*t*_: defect energy level relative to CB/VB and *N*_*bulk*_: bulk defect concentration. Subscripts *e* and *h* are electron and hole, respectively.

Properties	MoO_*x*_	NiO	Sb_2_Se_3_	CdS	*i*-ZnO	ITO
*d* (nm)	15	15	500 (TE). 1,000 (CSS)	70	35	200
*E*_*g*_ (eV)	3.85^*a*^	3.95^*a*^	1.17^*a*^	2.72^*a*^	3.37^*b*^	3.72^*c*^
*χ* (eV)	2.20^*e*^	1.46^*f*^	4.15^*g*^	4.70^*c*^	4.70^*c*^	4.50^*days*^
*ɛ*/*ɛ*_0_	10.0^*e*^	11.9^*f*^	14.4^*g*^	9.0^*b*^	9.0^*b*^	9.4^*days*^
*N*_*C*_ (cm^−3^)	2.2 × 10^18*e*^	2.2 × 10^18*f*^	2.2 × 10^18*g*^	2.1 × 10^18*b*^	1.8 × 10^19*b*^	4.0 × 10^19*c*^
*N*_*V*_ (cm^−3^)	1.8 × 10^19*e*^	1.8 × 10^19*f*^	1.8 × 10^19*g*^	1.7 × 10^19*b*^	2.4 × 10^18*b*^	1.0 × 10^18*c*^
*μ*_*e*_ (cm^2^/Vs.)	30^*e*^	2.8*f*	100^*g*^	160^*b*^	200^*b*^	30^*b*^
*μ*_*h*_ (cm^2^/Vs.)	2.5^*e*^	2.8^*f*^	25^*g*^	15^*b*^	93^*b*^	5^*b*^
*N*_*A*/*D*_ (cm^−3^)	D:3 × 10^16*e*^	A:3 × 10^18*f*^	A:1 × 10^14*h*^ (TE). A:1 × 10^16*i*^ (CSS)	D:1 × 10^17*b*^	D:1 × 10^18*b*^	D:1 × 10^21*b*^
Defects at Sb_2_Se_3_/CdS interface (Gaussian distribution throughout interface)						
*N*_*int*_ (cm^−3^)			D: varied	A: varied		
*σ*_*e*_ (cm^2^)			10^–13^	10^–15^		
*σ*_*h*_ (cm^2^)			10^–15^	10^–13^		
Bulk Sb_2_Se_3_ defects (Gaussian distribution throughout bulk)						
*N*_*bulk*_ (cm^−3^)			D: 2.6 × 10^16*j*^	A: 5.0 × 10^15*b*^		
*E*_*t*_ (eV)			0.62^*j*^	1.20^*b*^		
*σ*_*e*_ (cm^2^)			10^–13^	10^–17^		
*σ*_*h*_ (cm^2^)			10^–15^	10^–13^		

^*a*^Experimentally determined from UV-VIS, measurements.

^*b*^Reference (Kanevce et al., 2015).

^*c*^Reference (Erkan et al., 2016).

^*d*^Reference (Kartopu et al., 2019).

^*e*^Reference (Ni et al., 2019).

^*f*^Reference (Casas et al., 2017).

^*g*^Reference (Maurya and Singh, 2021).

^*h*^Experimentally determined from capacitance-voltage *C-V* measurements.

^*i*^Reference (Phillips et al., 2019).

^*j*^Reference (Chen and Tang, 2020).

**FIGURE 15:**
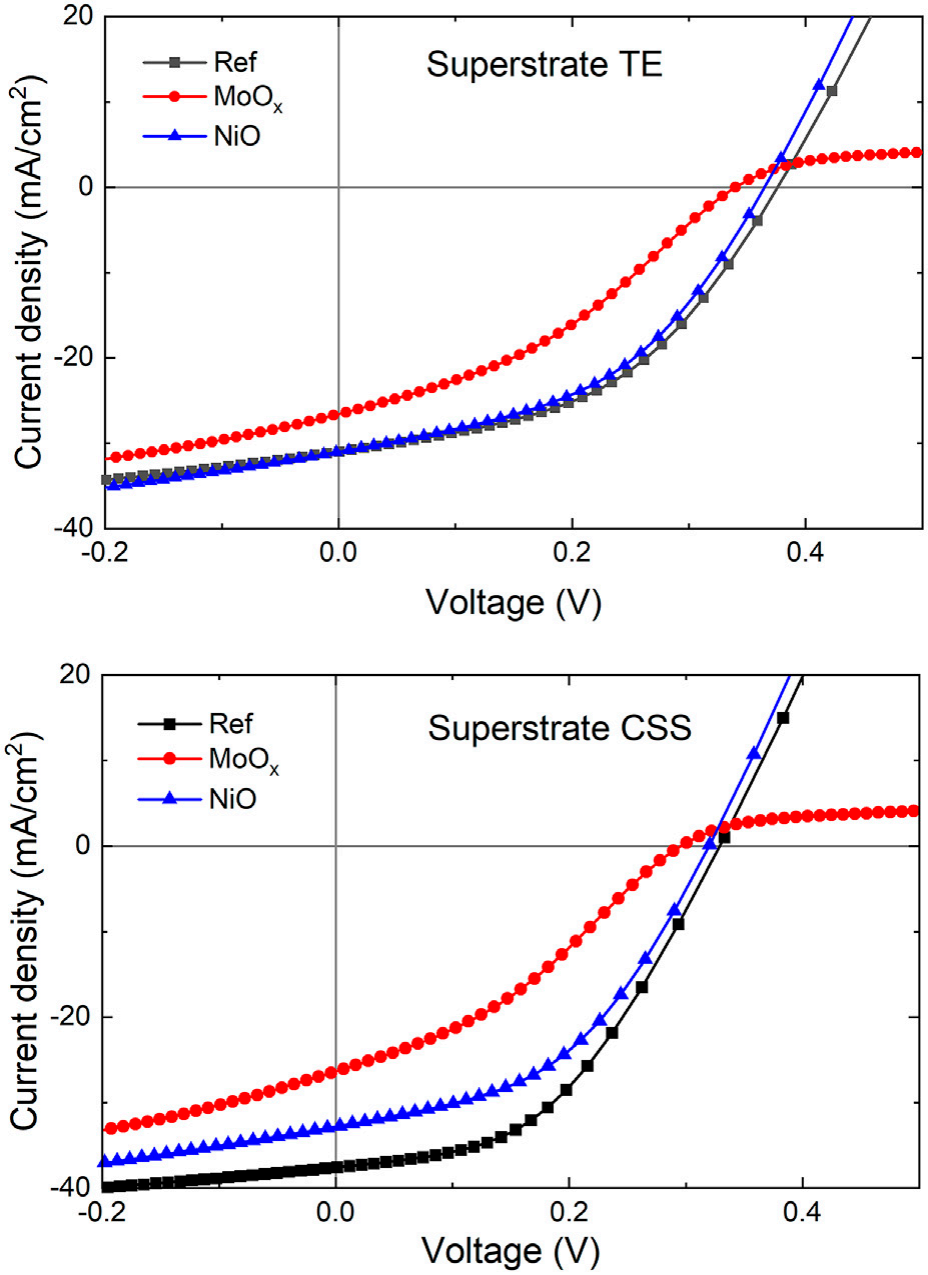
*J-V* curves for simulated TE and CSS Sb_2_Se_3_ devices with HTLs in superstrate configuration. Roll-over behaviour is evident in both TE and CSS devices with MoO_*x*_ HTL indicating a carrier transport barrier at the back contact.

In addition, it is worth noting actual superstrate devices which incorporate a MoO_*x*_ HTL under-perform in relation to standard simulated superstrate devices (see Figure 8). This decrease in performance is not observed in the fabricated substrate Sb_2_Se_3_ solar cells with a MoO_*x*_ HTL. This discrepancy can be accounted for by different processing conditions applied during deposition of substrate and superstrate devices. During deposition of Sb_2_Se_3_ layer on SLG/Mo/HTL substrate, the substrate temperature is maintained at 300°C which is sufficient to crystallise the MoO_*x*_ film, see Supplementary Materials S4. The crystallised MoO_*x*_ film consists of a mixture of MoO_2_, MoO_3_ and intermediate reduced oxide phases. The phase composition affects the electronic and optical properties of the MoO_*x*_ film, with MoO_2_ content lowering the resistivity, transmittance and bandgap (Inzani et al., 2017). Simulations also show a roll-over in the *J-V* curves for superstrate Sb_2_Se_3_ devices in both configurations (see Figure 15), indicating the presence of a barrier to carrier transport at the back contact seen in simulated energy band alignments as previously discussed (Figure 4).

## 4 Conclusion

Numerical simulations of standard planar superstrate and substrate Sb_2_Se_3_ solar cells along with the effect of incorporating MoO_*x*_ and NiO HTLs, demonstrated an increase in device efficiency for cells with a HTL which was achieved by an increase in *J*_*sc*_ for both substrate and superstrate device configurations. Both HTLs have high bandgaps and low electron affinities compared to Sb_2_Se_3_ absorber which manifests as a large barrier for electrons at the metallic back electrode and facilitates hole extraction. However, a roll-over effect was seen in the simulated *J-V* curve of the substrate device with MoO_*x*_ HTL, suggesting a current-blocking barrier at the back contact caused by non-optimal energy band alignment. Material characterisation of the HTL materials deposited by E-beam evaporation at room temperature revealed MoO_*x*_ formed an amorphous layer while NiO crystallised in cubic crystal orientation. 15 nm thick HTLs were incorporated into superstrate/substrate solar cells with Sb_2_Se_3_ absorbers deposited by thermal evaporation and close-space sublimation. For CSS superstrate solar cells with NiO HTL, device efficiency was enhanced by a 40% increase in *J*_*sc*_ compared to reference and MoO_*x*_ based devices. TE superstrate cells incorporating NiO as HTL also demonstrated improved efficiencies achieved by higher *V*_*oc*_ and *J*_*sc*_. In the superstrate TE cells with MoO_*x*_ HTL, *J*_*sc*_ was severely inhibited which is attributed to MoO_*x*_ forming a more resistive layer due to its amorphous nature. Conversely, the presence of a MoO_*x*_ or NiO HTL in substrate CSS-deposited Sb_2_Se_3_ solar cells reduced device performance which is linked to lower average *R*_*sh*_ observed in these cells. Optimisation of HTL thickness and/or re-optimisation of the absorber deposition could potentially alleviate this issue. Simulations reveal a connection between the WF of the Mo metal back contact and substrate device performance. For an experimentally determined Mo WF of 4.6 eV, all device *J-V* characteristics are significantly reduced, whereas substrate devices with NiO HTL are only marginally affected. In addition, XRD analysis of TE Sb_2_Se_3_ films with NiO HTL revealed a templating effect on Sb_2_Se_3_ crystal orientation where detrimental (020)/(120) crystal planes were eliminated and preferred (211)/(221) planes increased in intensity which resulted in increased device performance of substrate Sb_2_Se_3_ solar cells. NiO shows more promise as a HTL in Sb_2_Se_3_ PV devices, and crucially can act as a templating layer when the Sb_2_Se_3_ deposition method does not already impart the desired structure, as is often the case with TE devices.

## Data Availability

Data is available *via* this link: https://figshare.com/projects/Routes_to_Increase_Performance_for_Antimony_Selenide_Solar_Cells_using_Inorganic_Hole_Transport_Layers/140140.
