# Supplementary figures and images for: Routes to increase performance for antimony selenide solar cells using inorganic hole transport layers
Source: Front Chem. 2022 Sep 26;10:954588. doi: 10.3389/fchem.2022.954588 (PMC9548559; doi:10.3389/fchem.2022.954588)

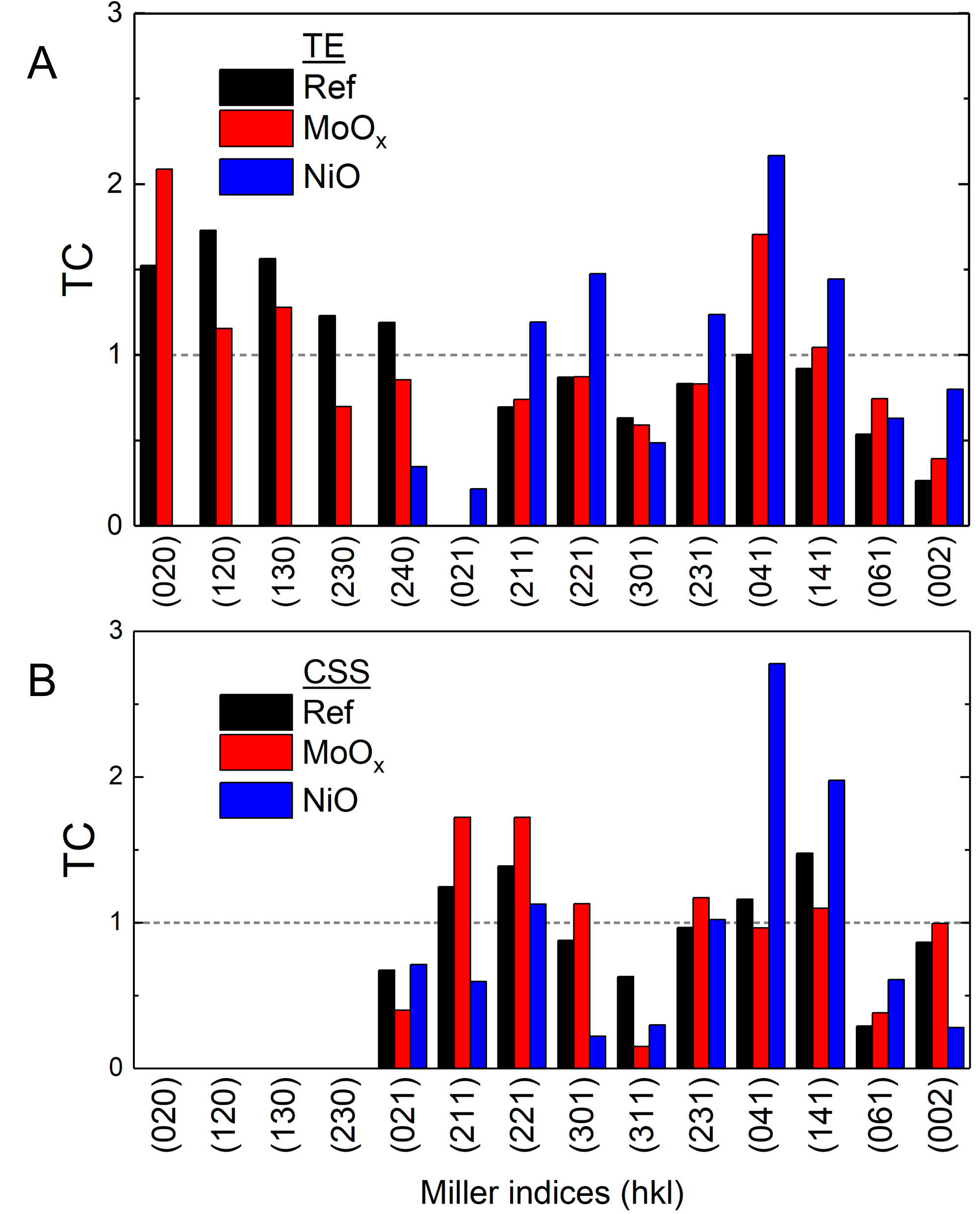

Supplement: Supplementary file 1 [file Image3.JPEG]

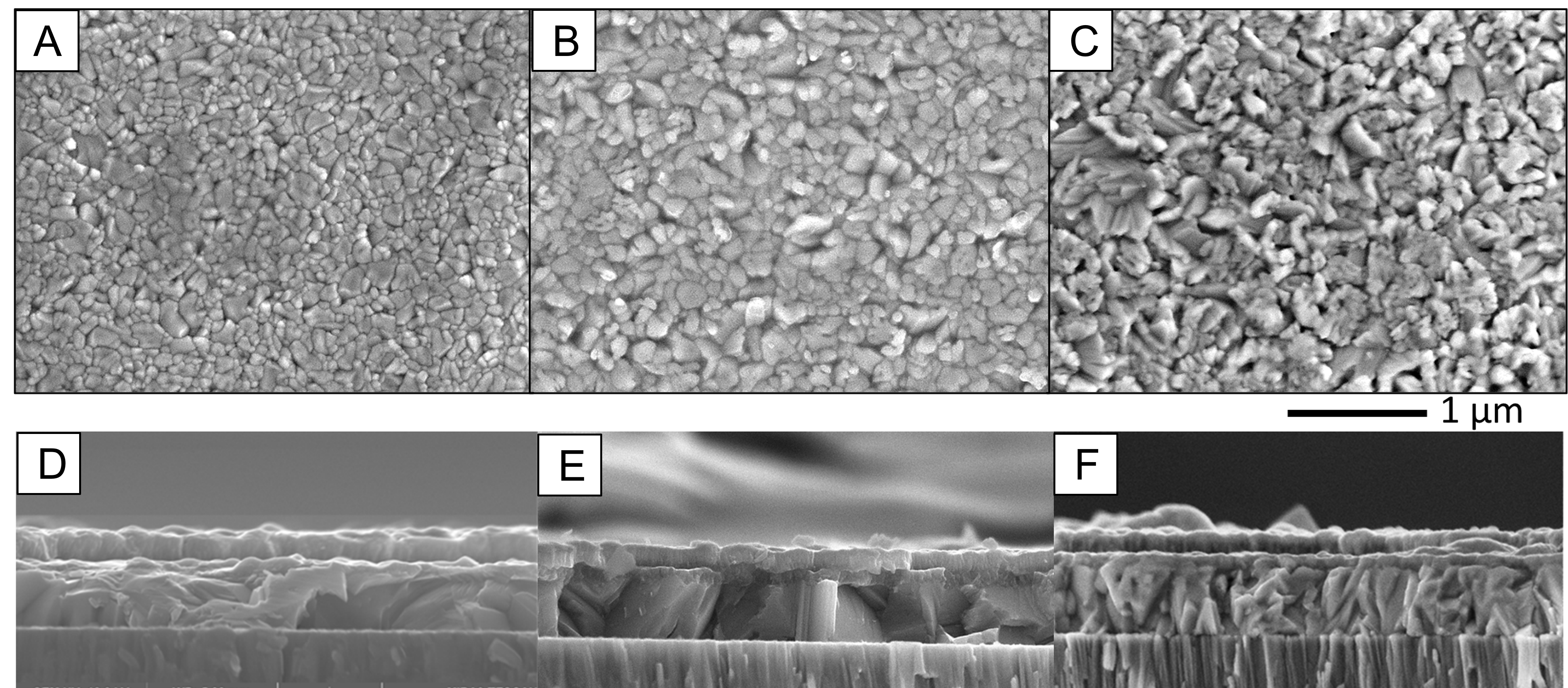

Supplement: Supplementary file 2 [file Image1.JPEG]

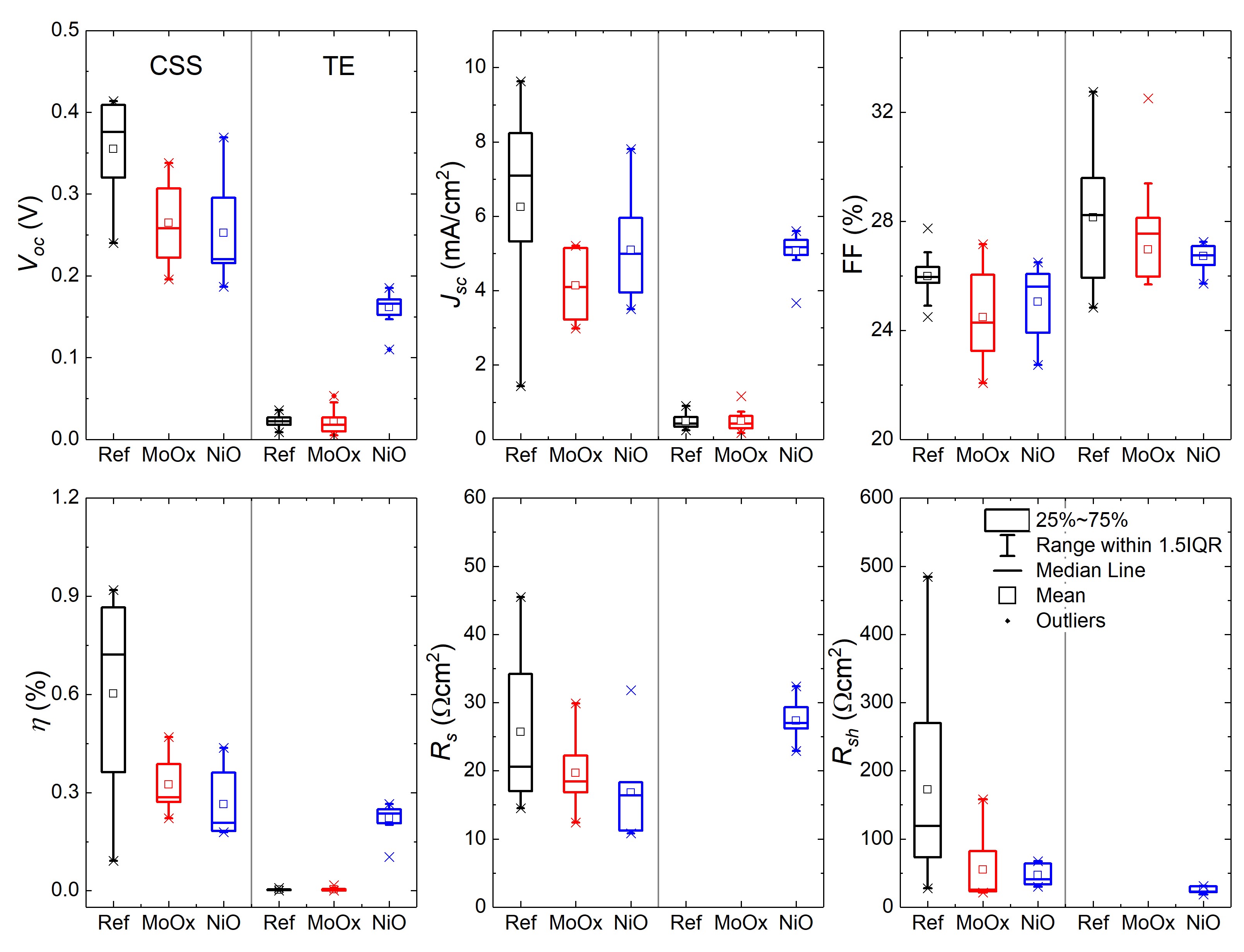

Supplement: Supplementary file 3 [file Image4.JPEG]

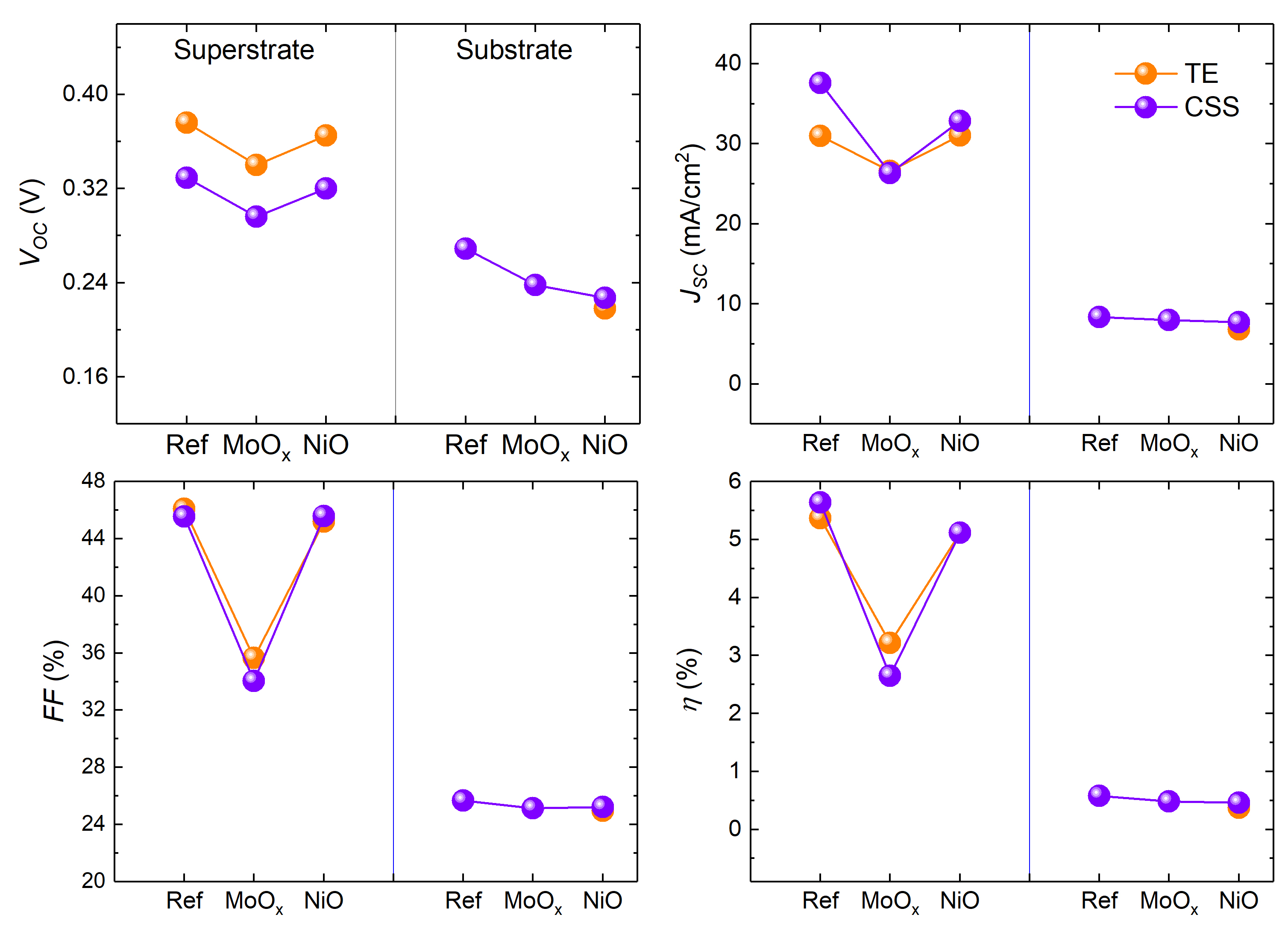

Supplement: Supplementary file 4 [file Image5.JPEG]

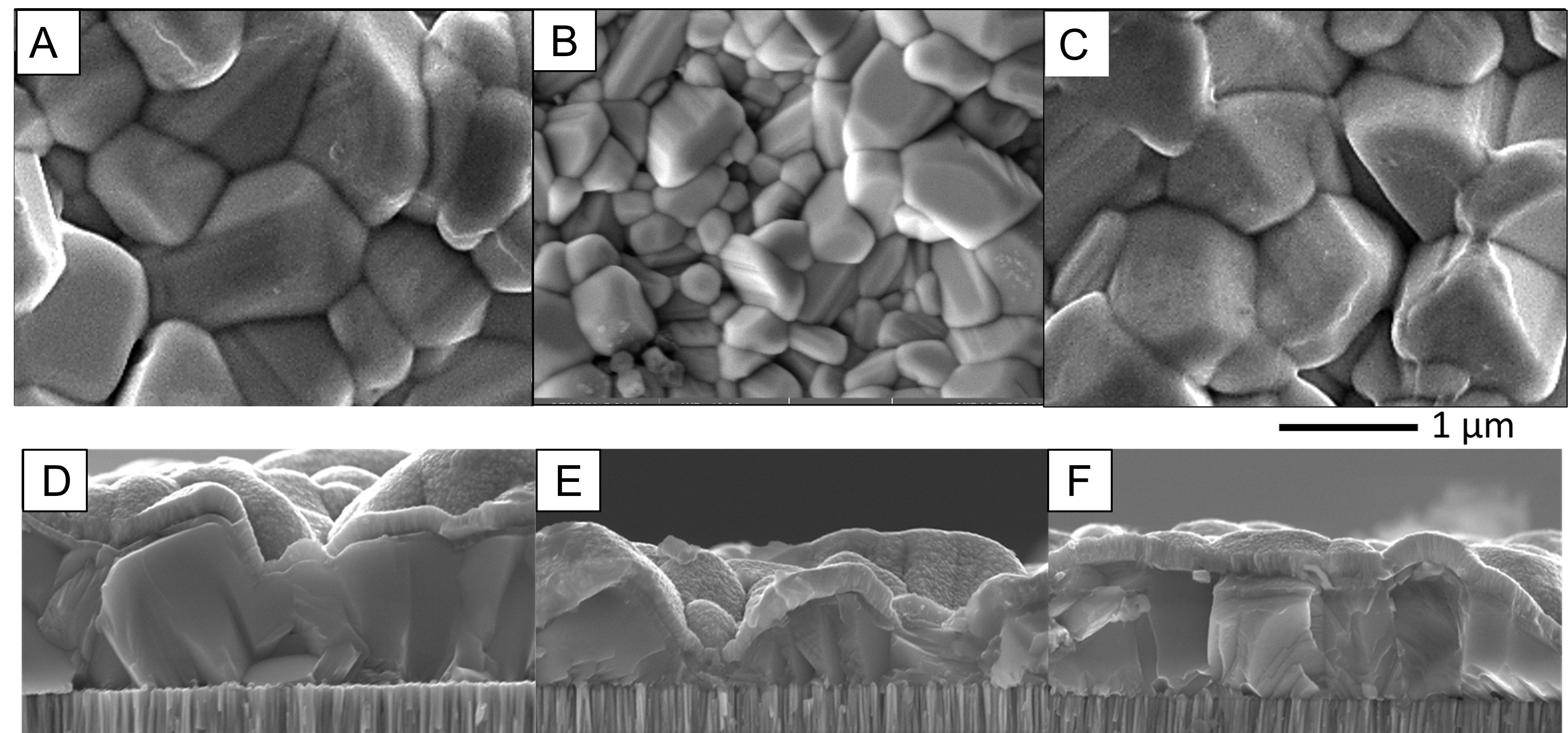

Supplement: Supplementary file 6 [file Image2.TIFF]

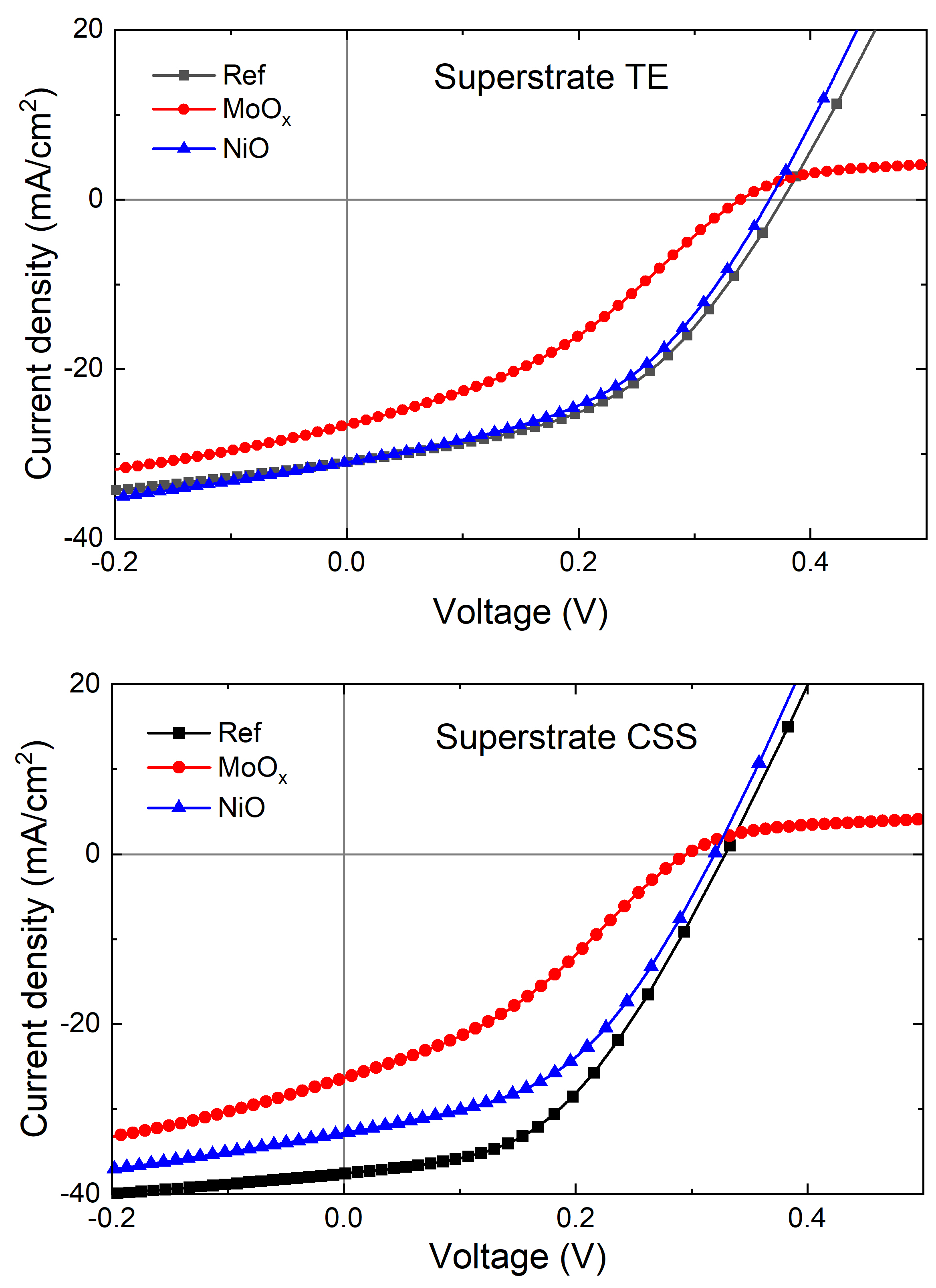

Supplement: Supplementary file 7 [file Image6.JPEG]
