# Supplementary material for: Routes to increase performance for antimony selenide solar cells using inorganic hole transport layers
Source: Front Chem. 2022 Sep 26;10:954588. doi: 10.3389/fchem.2022.954588 (PMC9548559; doi:10.3389/fchem.2022.954588)
Supplement: Supplementary file 5 [file DataSheet1.pdf]

Supplementary Material

1 SUPPLEMENTARY DATA

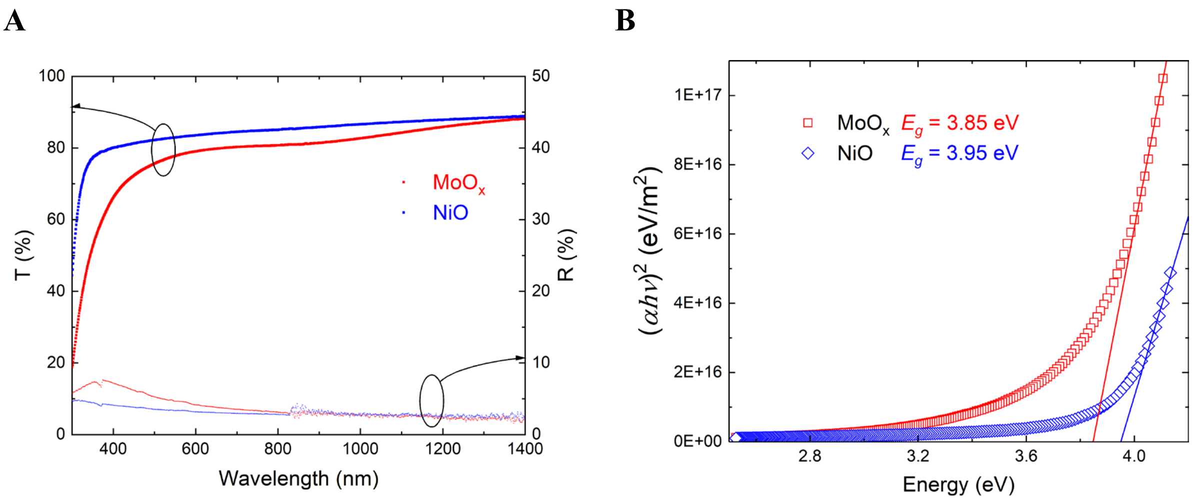

Figure S1: (**A**) Transmittance and reflectance of 15 nm evaporated MoO_x and NiO films on glass and (**B**) Tauc plots of the absorption spectra of the same films, showing the wide band gap.

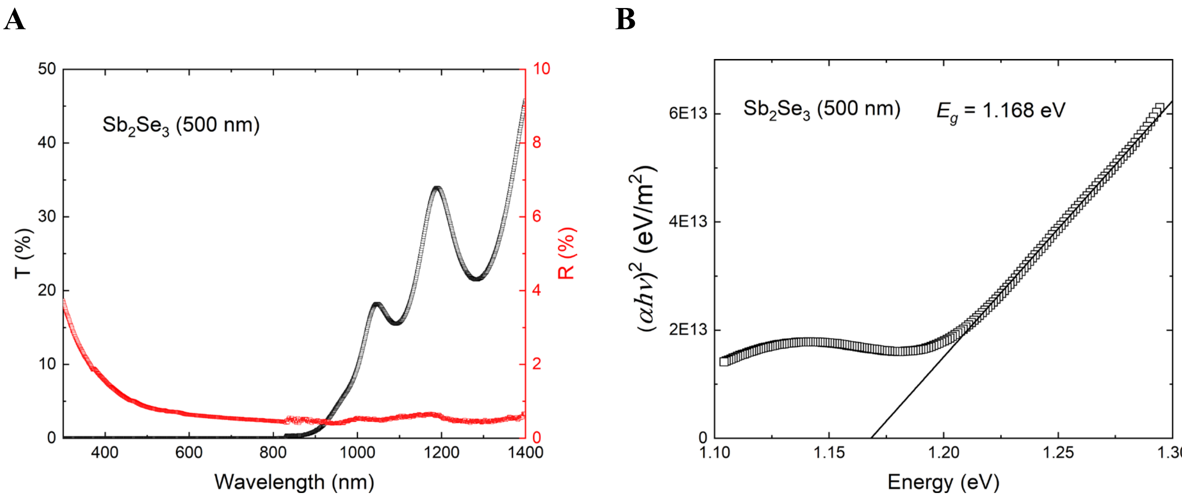

Figure S2: (**A**) Transmission and reflectance spectra for 500 nm thermally evaporated Sb_2Se_3 films on glass and (**B**) Tauc plot of the absorption spectrum of 500 nm thermally evaporated Sb_2Se_3 films on glass, showing the band gap.

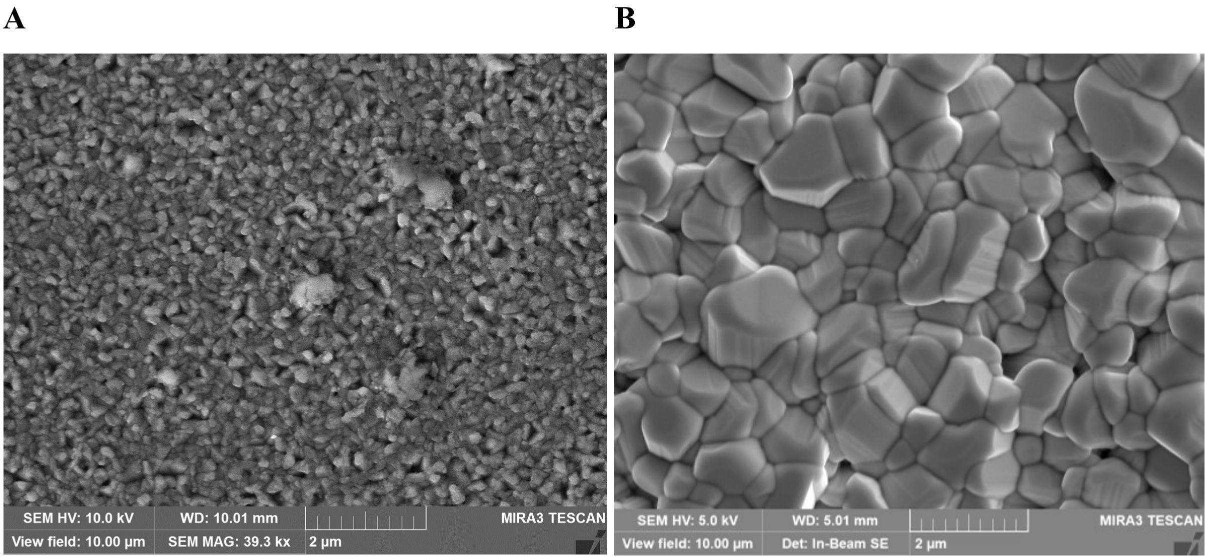

Figure S3: SEM image of Sb_2Se_3 film grown by (**A**) thermal evaporation and (**B**) closed space sublimation on SLG/ITO/CdS superstrate showing presence of pinholes across the thin film.

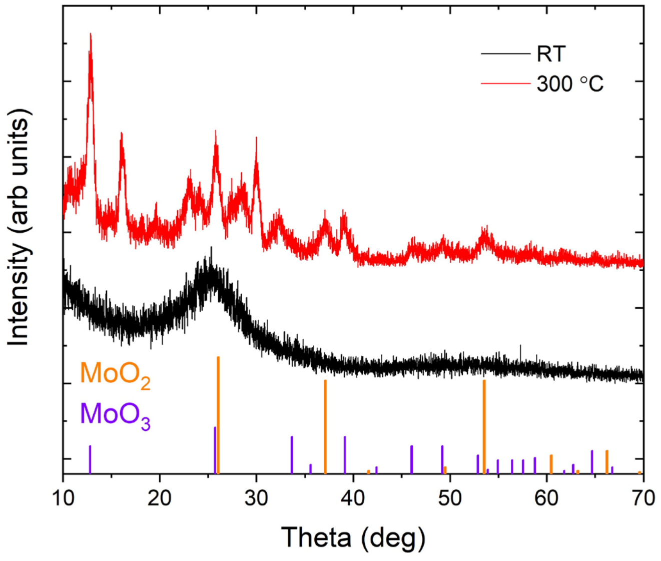

Figure S4: XRD patterns of the molybdenum oxide films deposited at room temperature and heated to 300°C, with the reference diffraction patterns MoO_2 (PDF 00-032-0671) and MoO_3 (00-005-0337). Additional peaks relate to other reduced molybdenum oxides.
